# USP7 negatively controls global DNA methylation by attenuating ubiquitinated histone-dependent DNMT1 recruitment

**DOI:** 10.1038/s41421-020-00188-4

**Published:** 2020-08-24

**Authors:** Jialun Li, Ruiping Wang, Jianyu Jin, Mengmeng Han, Zhaosu Chen, Yingying Gao, Xueli Hu, Haijun Zhu, Huifang Gao, Kongbin Lu, Yanjiao Shao, Cong Lyu, Weiyi Lai, Pishun Li, Guang Hu, Jiwen Li, Dali Li, Hailin Wang, Qihan Wu, Jiemin Wong

**Affiliations:** 1 Shanghai Key Laboratory of Regulatory Biology, Institute of Biomedical Sciences and School of Life Sciences, East China Normal University, Shanghai, 200241 China; 2 Joint Center for Translational Medicine, Fengxian District Central Hospital, 6600th Nanfeng Road, Fengxian District, Shanghai, 201499 China; 3 College of Education, Wenzhou University, Wenzhou, Zhejiang 325035 China; 4 State Key Laboratory of Environmental Chemistry and Ecotoxicology, Research Center for Eco-Environmental Sciences, Chinese Academy of Sciences, Beijing, 100085 China; 5 Epigenetics and Stem Cell Biology Laboratory, National Institute of Environmental Health Sciences, RTP, Durham, NC 27709 USA; 6 Key Laboratory of Reproduction Regulation of NPFPC, SIPPR, IRD, Fudan University, Shanghai, 200032 China

**Keywords:** Cancer genetics, DNA methylation

## Abstract

Previous studies have implicated an essential role for UHRF1-mediated histone H3 ubiquitination in recruiting DNMT1 to replication sites for DNA maintenance methylation during S phase of the cell cycle. However, the regulatory mechanism on UHRF1-mediated histone ubiquitination is not clear. Here we present evidence that UHRF1 and USP7 oppositely control ubiquitination of histones H3 and H2B in S phase of the cell cycle and that DNMT1 binds both ubiquitinated H3 and H2B. USP7 knockout markedly increased the levels of ubiquitinated H3 and H2B in S phase, the association of DNMT1 with replication sites and importantly, led to a progressive increase of global DNA methylation shown with increased cell passages. Using DNMT3A/DNMT3B/USP7 triple knockout cells and various DNA methylation analyses, we demonstrated that USP7 knockout led to an overall elevation of DNA methylation levels. Mechanistic study demonstrated that USP7 suppresses DNMT1 recruitment and DNA methylation through its deubiquitinase activity and the interaction with DNMT1. Altogether our study provides evidence that USP7 is a negative regulator of global DNA methylation and that USP7 protects the genome from excessive DNA methylation by attenuating histone ubiquitination-dependent DNMT1 recruitment.

## Introduction

In mammals, DNA methylation at cytosine is an epigenetic modification required for embryonic development, transcriptional regulation, heterochromatin formation, X-inactivation, imprinting, transposon silencing, and genome stability^1–3^. DNA methylation in mammalian cells is catalyzed by three active DNA methyltransferases, namely DNMT3A, DNMT3B, and DNMT1. Although in principle the level of DNA methylation in a given cell is determined by a coordinated activity of three DNMTs and active demethylation by enzymes such as TET family proteins^4,5^, DNMT1 has been shown to play a central role in maintaining DNA methylation patterns in differentiated cells^6^. While predominantly as a maintenance enzyme, previous studies have also detected DNMT1 de novo activity both in vitro and in vivo^7–10^. However, how DNMT1’s activity is regulated in mammalian cells to ensure stable inheritance of DNA methylation remains incompletely understood.

Accumulative studies have established a key role for UHRF1 (also known as ICBP90 in humans and NP95 in mice) and its catalyzed histone H3 ubiquitination in targeting DNMT1 to DNA replication fork^11–14^. As a multi-structural and functional domain nuclear protein, UHRF1 specifically binds replication fork in S phase by a coordinated binding of hemi-methylated CpGs (products of newly replicated DNA) via a unique SRA domain^15–17^ and the histone H3 tail without or with di- or tri-methylated K9 (H3K9me2/3) via its Tandem Tudor domain and a PHD domain^18–28^. A recent study^29^ provides evidence that UHRF1 can also associate with replication fork by binding methylated DNA ligase 1. Although the initial work suggested that replication fork-associated UHRF1 may recruit DNMT1 via a direct protein–protein interaction^11,12^, recent studies provide compelling evidence that UHRF1’s ubiquitin E3 ligase activity is required for DNMT1 recruitment^13,14^ and ubiquitinated H3 not only binds DNMT1 but also activates DNMT1 enzymatic activity^30^. So far UHRF1 has been shown to ubiquitinate H3 N-terminal tail at multiple sites^13,14,31^, whereas DNMT1 binds ubiquitinated H3 (in particularly di-ubiquitinated H3) through an ubiquitin-binding domain (UIM) within DNMT1’s replication foci targeting sequence (RFTS)^14,30^. While current evidence highlights a central role for the interaction between ubiquitinated H3 and DNMT1 in DNA maintenance methylation, how the level of ubiquitinated H3 is regulated is poorly understood. Furthermore, it is not known whether UHRF1 also ubiquitinates other histones in S phase of the cell cycle. Similarly, it is not clear if DNMT1 also binds other ubiquitinated histones.

USP7 (ubiquitin specific protease 7, also known as HAUSP), is a deubiquitinase that has been previously shown to regulate the stability of various proteins, including p53 and MDM2, and plays roles in cell cycle, DNA repair, and epigenetic regulation^32–36^. Before it was reported to deubiquitinate H3, USP7 was known to deubiqutinate histone H2B^37^. Of note, USP7 has been repeatedly identified as a major interacting protein of DNMT1 and UHRF1, raising significant interest in its function in DNA methylation^38–43^. So far most studies support a role of USP7 in regulating protein stability of both UHRF1 and DNMT1^38–41^, However, how USP7 regulates DNA methylation is much less clear. In one study, USP7 was shown to stimulate DNMT1 activity in vitro and knockdown of USP7 reduced the levels of DNA methylation on several silenced genes^39^. In another study with Xenopus egg extracts, USP7 was shown to deubiqutinate H3 and depletion of USP7 caused a delay of DNA methylation kinetics^44^. Interestingly, it was also shown in this study that knockdown of USP7 in HeLa cells led to an enhanced DNMT1 recruitment to DNA replication sites, although whether knockdown of USP7 affected DNA methylation was not reported. In a more recent study, it was reported that acute USP7 knockout in MEFs or knockdown in H1299 cells affected neither the level of DNMT1 proteins nor global level of DNA methylation^45^. Thus, the exact role of USP7 and its regulated histone deubiquitination in DNA methylation needs to be further clarified.

In this study, we provide evidence that USP7 is a negative regulator of global DNA methylation. It is required to suppress ubiquitinated histone-dependent DNMT1 recruitment in S phase, which prevents excessive DNA methylation by DNMT1.

## Results

### UHRF1 and USP7 control dynamic ubiquitination of histones H3 and H2B in S phase of cell cycle

**Fig. 1:**
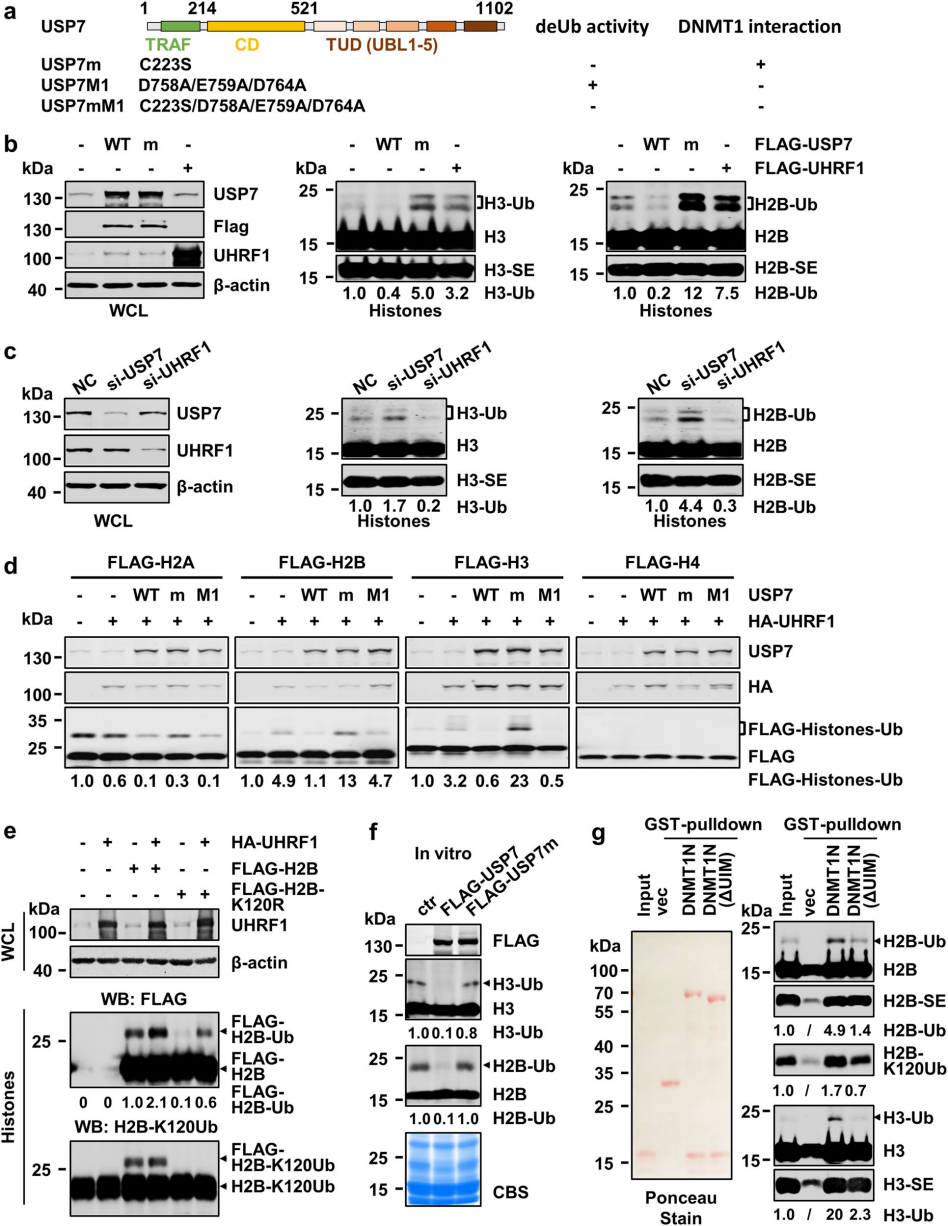
USP7 deubiquitinates UHRF1-mediated ubiquitination of H3 and H2B in S phase of the cell cycle. **a** Schematic diagram showing USP7 structural organization and mutants used in this study. **b** WB analysis showing that ectopically expressed USP7 and UHRF1 oppositely controlled ubiquitination on histones H2B and H3 in HEK293T cells. Note that USP7 catalyzed deubiquitination of H2B and H3 depending on its deubiquitinase activity. All cells were synchronized to S phase by aphidicolin treatment followed by release in fresh medium for 4 h. Core histones were prepared by acid extraction. Also shown are short exposure images of H3 (H3-SE) and H2B (H2B-SE). **c** WB analysis showing that knockdown of USP7 or UHRF1 by siRNA oppositely affected H3 and H2B ubiquitination in HEK293T cells in S phase. **d** WB analysis showing that ectopically expressed histones were deubiquitinated by USP7 and ubiquitinated by UHRF1. Note that deubiquitination by USP7 is dependent on its deubiquitinase activity. **e** UHRF1 ubiquitinated H2B at site(s) other than K120. HA-UHRF1 and FLAG-H2B or H2B-K120R mutant were co-expressed in HEK293T cells. The core histones were prepared by acid extraction and subjected to WB analysis using either anti-FLAG or anti-H2B-K120Ub antibody as indicated. **f** WB analysis showing that purified USP7 but not USP7m mutant deubiquitnated H2B and H3 in vitro. **g** In vitro pull-down assay showing DNMT1 preferentially bound both ubiquitinated H3 and H2B. The core histones were prepared from HEK293T cells transfected with FLAG-H3 or FLAG-H2B together with UHRF1 and synchronized to the S phase of cell cycle as above. WB was performed with anti-FLAG antibody.

To investigate the role of USP7 in DNA methylation, we first analyzed if USP7 regulates H3 ubiquitination in S phase of cell cycle. As a pilot test, we ectopically overexpressed wild-type, a deubiquitinase-deficient USP7 C223S mutant (USP7m) (Fig. 1a)^32^ and UHRF1 in HEK293T cells and synchronized cells to S phase by aphidicolin treatment followed by a 4 h-release in fresh medium. The ectopic overexpression of USP7 was confirmed by western blotting analysis (WB) using both anti-FLAG and anti-USP7 antibodies (Fig. 1b, left panel). Subsequent WB analysis revealed that ectopic expression of USP7 markedly reduced the levels of two higher molecular weight bands detected by a H2B-specific antibody (Fig. 1b, right panel). Similarly, ectopic expression of USP7 also markedly reduced the levels of two higher molecular weight bands detected by a H3-specific antibody (Fig. 1b, middle panel). Notably, these higher molecular H2B and H3 bands were increased upon ectopic expression of UHRF1 and displayed molecular weights around 25 kD, suggesting these are most likely mono-ubiquitinated H2B or H3 with ubiquitin at two different sites or different histone variants. The observed deubiquitination of both H2B and H3 by wild-type USP7 is consistent with reported USP7 H2B deubiquitinase activity^37^ and H3 deubiquitinase activity^45^. Interestingly, expression of the deubiquitinase activity-deficient USP7m markedly elevated H3 and H2B ubiquitination, suggesting this mutant acted as a dominant negative to inhibit deubiquitination of H3 and H2B by endogenous USP7 (Fig. 1b). Of note, we observed that expression of UHRF1 not only elevated the level of H3 ubiquitination but also H2B ubiquitination (Fig. 1b), suggesting that UHRF1 ubiquitinates both H3 and H2B in the S phase of cell cycle.

To test if endogenous USP7 and UHRF1 oppositely regulate H2B and H3 ubiquitination in S phase, we knocked down USP7 or UHRF1 by siRNA in HEK293T cells and synchronized cells to S phase (Fig. 1c). Subsequent WB analysis revealed that knockdown of USP7 consistently led to an increased level of H2B and H3 ubiquitination (Fig. 1c). On the other hand, knockdown of UHRF1 by siRNA resulted in a clear reduction of both ubiquitinated H3 and ubiquitinated H2B (Fig. 1c). Together these results provide evidence that USP7 and UHRF1 oppositely regulate H3 and H2B ubiquitination in S phase of cell cycle.

To confirm and systematically examine UHRF1’s ability to catalyze histone ubiquitination, we co-expressed UHRF1 with each FLAG-tagged histone protein in HEK293T cells and evaluated ubiquitination on FLAG-histones by WB analysis of acid-extracted core histones. We observed that UHRF1 promoted not only FLAG-H3 (3.2-fold) but also FLAG-H2B ubiquitination (4.9-fold) (Fig. 1d). Under the same condition, UHRF1 did not affect FLAG-H2A ubiquitination and no ubiquitination on FLAG-H4 was observed (Fig. 1d). It is noteworthy that while two distinct ubiquitinated populations were observed for endogenous H2B and H3 (Fig. 1b, c), only one major ubiquitinated band was, respectively, observed for ectopically expressed FLAG-H2B and FLAG-H3 (Fig. 1d). The reason for this difference is not clear, but could be related to histone variants and partial degradation of endogenous histone proteins. We next tested if co-expression of USP7 is able to diminish UHRF1-catalyzed ubiquitination on FLAG-H2B and H3. As shown in Fig. 1d, co-expression of USP7 markedly diminished UHRF1-catalyzed H3 and H2B ubiquitination. The ability to deubiquitinate histones was dependent on USP7’s deubiquitinase activity but not its interaction with DNMT1, as a D758A/E759A/D764A mutant (USP7M1) defective in DNMT1 interaction^42^ but not USP7m was able to deubiquitinate H2B and H3 (Fig. 1d and Supplementary Fig. S1a). The enhanced H3 and H2B ubiquitination with USP7m again suggested a dominant negative effect of this mutant (Fig. 1d). We further confirmed that UHRF1 and USP7 can dynamically regulate H3 and H2B ubiquitination and the identities of ubiquitinated H2B and H3 by using Myc-tagged ubiquitin (Supplementary Fig. S1b).

We also tested if UHRF1 ubiquitinates H2B at K120, a well characterized ubiquitination site on H2B in mammalian cells. As shown in Fig. 1e, UHRF1 was able to ubiquitinate FLAG-H2B with K120R mutation. Furthermore, WB analysis using an H2B-K120Ub-specific antibody revealed that ectopic overexpression of UHRF1 did not appear to increase the level of H2B-K120Ub. Taken together, UHRF1 does not appear to catalyze H2B ubiquitination at K120 site. However, we have yet to determine the exact site(s) on which UHRF1 catalyzes H2B ubiquitination.

We also tested if USP7 directly deubiquitinates H3 and H2B by in vitro deubiquitination assay. As shown in Fig. 1f, recombinant USP7 but not the enzymatic inactive USP7m mutant was able to deubiquitinate both H3 and H2B prepared by acid extraction.

Altogether, these results suggest that UHRF1 and USP7 can oppositely control not only H3 but also H2B ubiquitination in S phase of cell cycle.

### DNMT1 also binds ubiquitinated H2B

UHRF1-catalyzed H3 ubiquitination is believed to play a critical role in recruiting DNMT1 to replication fork. Our finding that UHRF1 and USP7 oppositely control both H3 and H2B ubiquitination in S phase of cell cycle prompted us to test if DNMT1 also binds ubiquitinated H2B. We fused the N-terminal RFTS-containing region of DNMT1, without or with deletion of the UIM required for binding of ubiquitinated H3^14^, to GST and prepared the recombinant proteins GST-DNMT1-N and GST-DNMT1-N-ΔUIM from *E. coli*. We also ectopically expressed UHRF1 in HEK293T cells and synchronized cells to S phase to enrich for ubiquitinated histones. Core histones were then prepared by acid extraction and subjected to GST pull-down. The results in Fig. 1g showed that ubiquitinated H3 was significantly enriched by GST-DNMT1-N (~20-fold), but not GST-DNMT1-N-ΔUIM, a result consistent with previous observation^14^. Importantly, we found that like ubiquitinated H3, ubiquitinated H2B was also enriched, although to a less extent (4.9-fold), by GST-DNMT1-N, but not GST-DNMT1-N-ΔUIM. Thus, our data suggest that not only can UHRF1 and USP7 dynamically regulate H3 and H2B ubiquitination, DNMT1 binds both ubiquitinated histones H3 and H2B.

### USP7 knockout results in elevated H3 and H2B ubiquitination in S phase of cell cycle

**Fig. 2:**
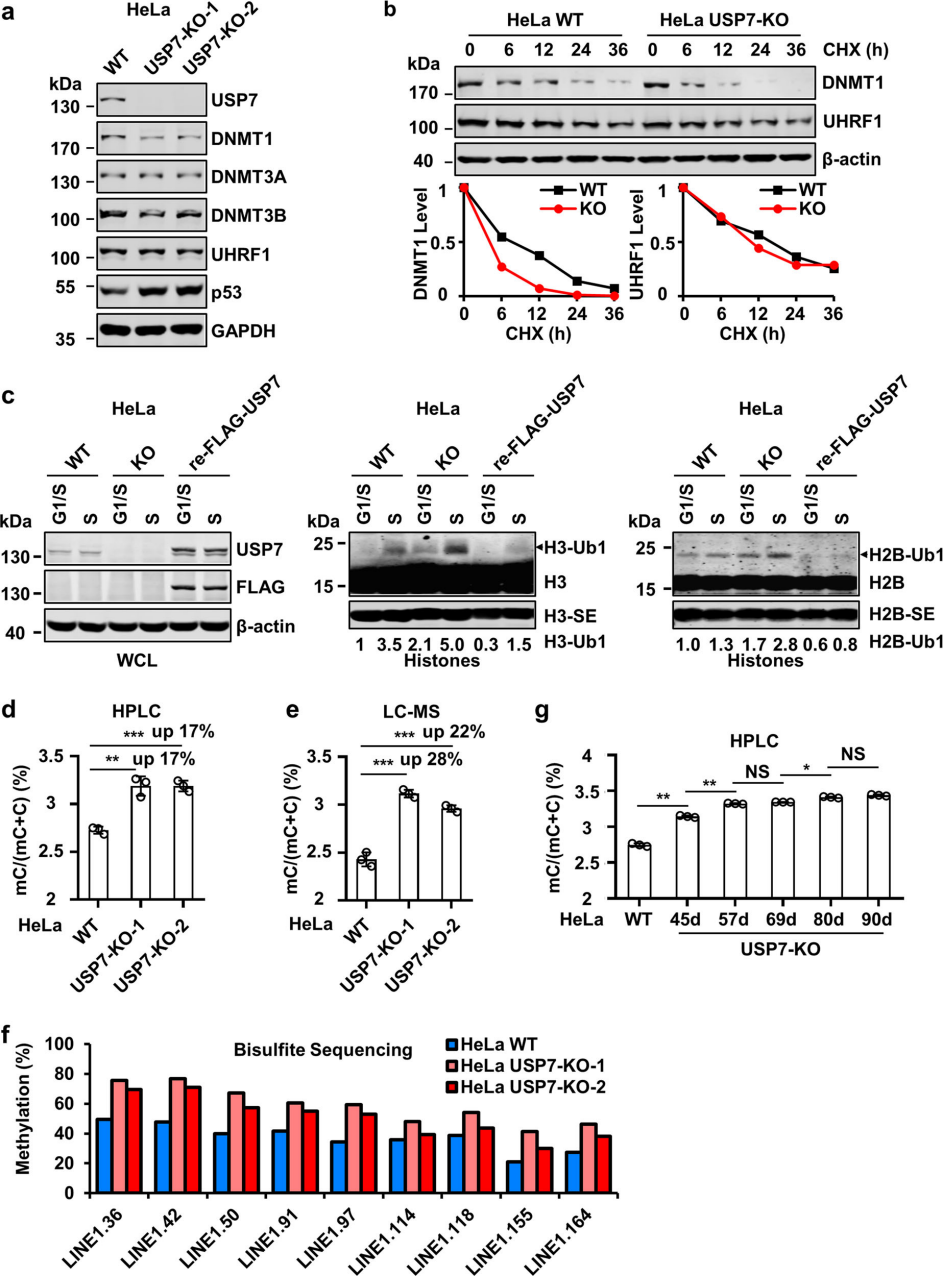
USP7 knockout results in increased DNA methylation in HeLa cells. **a** WB analysis showing the levels of key proteins involved in DNA methylation and p53 in control and two independent USP7-KO HeLa cell lines. **b** Analysis of DNMT1 and UHRF1 protein stability in control and USP7-KO HeLa cells. The cells were treated with cycloheximide (100 μg/mL) in culture medium for various times as indicated. The WB data were quantified by ImageJ and shown as relative levels to control (0 h) in the lower panel. **c** WB analysis showing USP7 controlled both H3 and H2B ubiquitination in S phase of the cell cycle. All cells were synchronized to G1/S boundary and S phase as above and core histones were prepared by acid extraction. Also shown are short exposure images of H3 (H3-SE) and H2B (H2B-SE). **d** The levels of genomic DNA methylation (mC) in control and USP7-KO HeLa cells determined by HPLC. ***P ≤ 0.001; **P ≤ 0.01. **e** The levels of genomic DNA methylation (mC) in control and USP7-KO HeLa cells determined by LC-MS. ***P ≤ 0.001. **f** The levels of DNA methylation for each indicated CpG site in LINE1 in control and USP7-KO HeLa cells determined by bisulfite next-generation sequencing. **g** The levels of genomic DNA methylation (mC) in control and USP7-KO HeLa cells with progressively increased culture time determined by HPLC. **P ≤ 0.01; *P ≤ 0.05; NS, not significant.

To further define the role of USP7 in regulation of histone ubiquitination and DNA methylation, we used CRISPR-Cas9 technique to knockout (KO) USP7 in HeLa cells (Supplementary Fig. S2a). Multiple individual clones were obtained and the knockout of USP7 was determined by both DNA sequencing and WB analysis (Supplementary Fig. S2b, c). WB analysis of two independent USP7-KO clones revealed that USP7 KO led to a mild reduction of DNMT1 and insignificant changes of the levels of DNMT3A, DNMT3B and UHRF1 proteins (Fig. 2a). USP7 KO in HeLa cells also resulted in an increase of p53 protein (Fig. 2a), in full agreement with previous studies showing increased p53 proteins in USP7^−/−^ HCT116 and mouse embryonic fibroblasts cells^33,34,46^. Although USP7 KO did not significantly affect the overall levels of DNMT1 and UHRF1 proteins in HeLa cells, subsequent protein stability assay using cycloheximide did reveal a reduced DNMT1 protein stability, a slightly reduced UHRF1 protein stability (Fig. 2b) and an increased p53 protein stability (Supplementary Fig. S2d). RT-PCR analysis revealed a significant increase of p21 transcript (consistent with increased p53) and moderate increase of DNMT1 and UHRF1 mRNAs (Supplementary Fig. S2e). Thus, our data revealed that loss of USP7 variably reduced DNMT1 and UHRF1 protein stability, presumably due to its deubiquitinase activity toward DNMT1 and UHRF1 as reported previously^38–41^. However, loss of USP7 did not significantly affect the overall levels of UHRF1 and DNMT1 proteins in HeLa, possibly due to an elevated level of DNMT1 and UHRF1 transcripts. We also observed that loss of USP7 led to reduced cell proliferation and increased population of G2 phase cells (Supplementary Fig. S2f), consistent with an increased p21 expression.

To examine the effect of USP7 KO on histone ubiquitination in S phase, we synchronized control and USP7-KO cells to G1/S boundary by aphidicolin treatment and S phase by release of aphidicolin-arrested cells in fresh medium for 4 h as above. WB analysis revealed that, when compared to G1/S cells, increased levels of ubiquitinated H3 (3.5-fold) and ubiquitinated H2B (1.3-fold) were observed for control S-phase cells (Fig. 2c). USP7 KO elevated the levels of ubiquitinated H3 by 2.1-fold and ubiquitinated H2B by 1.7-fold in G1/S cells. Notably, USP7 KO elevated the levels of ubiquitinated H3 and H2B in the S-phase 5.0- and 2.8-fold, respectively. Re-expression of FLAG-USP7 in USP7-KO cells markedly reversed the levels of ubiquitinated H2B and H3 both in G1/S and S phases (Fig. 2c), demonstrating that the observed elevated levels of histone ubiquitination were indeed due to loss of USP7. As UHRF1 is required for H3 and H2B ubiquitination in S phase (Fig. 1c), these results demonstrated that USP7 plays a role in downregulating UHRF1-catalyzed S-phase histone ubiquitination. Using a USP7-KO HCT116 cell line reported previously^35^, we confirmed that loss of USP7 also led to increased H2B and H3 ubiquitination in S phase of cell cycle (Supplementary Fig. S2g).

### Loss of USP7 results in increased global DNA methylation

Next, we prepared genomic DNA from both control and USP7-KO HeLa cell lines and quantitatively determined the levels of 5-methylcytosine (5mC) by quantitative high-performance liquid chromatography (HPLC) analysis. Notably, we found that loss of USP7 resulted in a substantial and statistically significant increase of global DNA methylation in two independent USP7-KO HeLa cell lines (3.19% mC in USP7-KO-1 and KO-2 vs. 2.73% mC in control, representing a ~17% increase) (Fig. 2d). Although differed in exact levels, increased DNA methylation in two independent USP7-KO cell lines was validated by independent quantitative measurement of 5mC by liquid chromatography-mass spectrometry (LC-MS) (Fig. 2e). To further substantiate this finding, we measured DNA methylation at the long interspersed nucleotide (LINE-1) elements, which represents global level of DNA methylation in mammalian cells, by bisulfite DNA sequencing of large number of clones by high throughput sequencing. The results in Fig. 2f confirmed increased DNA methylation for various LINE-1 CpG sites in two USP7-KO HeLa cell lines.

As increased DNA methylation was not observed in previous studies with short term knockdown of USP7^45^, we surmised whether the observed increase of DNA methylation in our study was associated with a long culture process of obtaining USP7-KO cell lines from individual single KO cells. Consistent with this idea and previous publications^38,39,45^, we found that knockdown of USP7 by treatment of siRNAs for 3 days did not significantly affect the global level of DNA methylation (Supplementary Fig. S2h, i). However, when DNA methylation was measured with USP7-KO cells cultured consecutively for various times, increased levels of DNA methylation were observed along with increased numbers of cell passages (Fig. 2g). Together these results demonstrated that, despite a moderate reduction in DNMT1 protein levels and a reduced DNMT1 protein stability, loss of USP7 in HeLa cells results in a progressively increased DNA methylation upon increased cell propagation, suggesting a negative regulatory role for USP7 in DNA methylation.

### USP7 also negatively regulates DNA methylation in HCT116 cells and mouse embryos

**Fig. 3:**
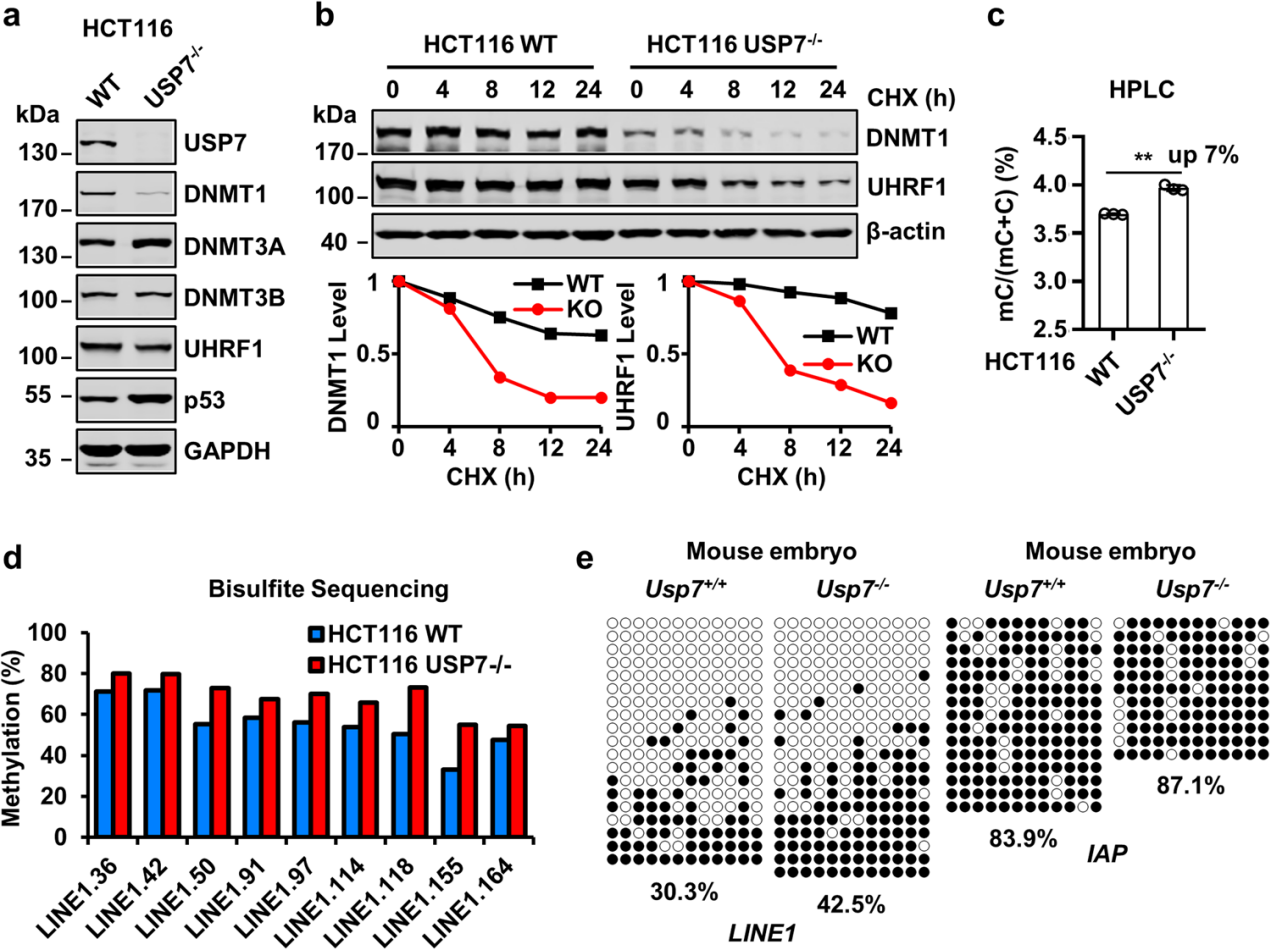
USP7 knockout results in increased DNA methylation in HCT116 cells and mouse embryos. **a** WB analysis showing the levels of key proteins in DNA methylation and p53 in control and USP7^−/−^ HCT116 cells. **b** Analysis of DNMT1 and UHRF1 protein stability in control and USP7^−/−^ HCT116 cells. The cells were treated with cycloheximide (100 μg/mL) in culture medium for various times as indicated. The WB data were quantified by ImageJ and shown as relative levels to control (0 h) in the lower panel. **c** The levels of genomic DNA methylation (mC) in control and USP7^−/−^ HCT116 cells determined by HPLC. ***P ≤ 0.001. **d** The levels of DNA methylation for each indicated CpG site in LINE1 in control and USP7^−/−^ HCT116 cells determined by bisulfite next-generation sequencing. **e** Bisulfite sequencing resulted comparing DNA methylation of LINE1 and IAP repetitive elements in control and *Usp7*^−/−^ mouse embryos.

To test further our surprising finding that USP7 negatively regulates DNA methylation, we next analyzed how loss of USP7 affects DNMT1 and UHRF1 protein stability and DNA methylation in HCT116 cells by using a previously reported USP7^−/−^ HCT116 cell line^33–36^. WB analysis revealed that loss of USP7 in HCT116 cells led to an ~70% reduction of DNMT1 protein and insignificant change of UHRF1 and DNMT3B and slightly increased DNMT3A (Fig. 3a). As reported previously^33,34^, loss of USP7 led to an increased level of p53 protein (Fig. 3a). Protein stability assay revealed a reduction of protein stability for both DNMT1 and UHRF1 in HCT116^−/−^ cells (Fig. 3b). Thus, in both HeLa and HCT116 cells USP7 appears to variably promote DNMT1 and UHRF1 protein stability. However, measurement of 5mC level by HPLC analysis revealed an increased level of DNA methylation in USP7^−/−^ HCT116 cells in comparison to control cells (Fig. 3c). Bisulfite sequencing analysis of LINE1 elements also confirmed an increase of DNA methylation in USP7^−/−^ HCT116 cells (Fig. 3d). Thus, despite a reduced level of DNMT1 and reduced stability of DNMT1 and UHRF1, loss of USP7 also results in an increased global DNA methylation in HCT116 cells.

A previous study demonstrated that USP7 is essential for mouse early embryonic development^46^. To test if USP7 negatively regulates DNA methylation in mice, we deleted the *Usp7* gene in in vitro-fertilized mouse embryos via CRISPR/Cas9 by using two guide RNAs^47^ (Supplementary Fig. S3a, b). The embryos injected with guide RNAs and Cas9 mRNA were cultured in vitro to morula stage and genomic DNA was prepared. The embryos with successful deletions of the *Usp7* gene was verified by PCR-based genotyping and sequencing (Supplementary Fig. S3b). As the limited amount of DNA obtained from a single embryo excluded measurement of 5mC by HPLC and LC-MS, we only carried out bisulfite sequencing analysis on *LINE1* and intracisternal A-type particle (*IAP*) repetitive sequences. As shown in Fig. 3e, a significant increased level of DNA methylation was detected for *LINE1* (from 30.3 to 42.5%, a more than 40% increase of DNA methylation), whereas a moderate increase of DNA methylation was observed for IAP upon deletion of *Usp*7. These results suggest that USP7 also negatively regulates global DNA methylation in mouse embryos. Taken together, we conclude that negative regulation of DNA methylation is likely a general function for USP7.

### USP7 negatively regulates DNMT1-mediated DNA methylation

**Fig. 4:**
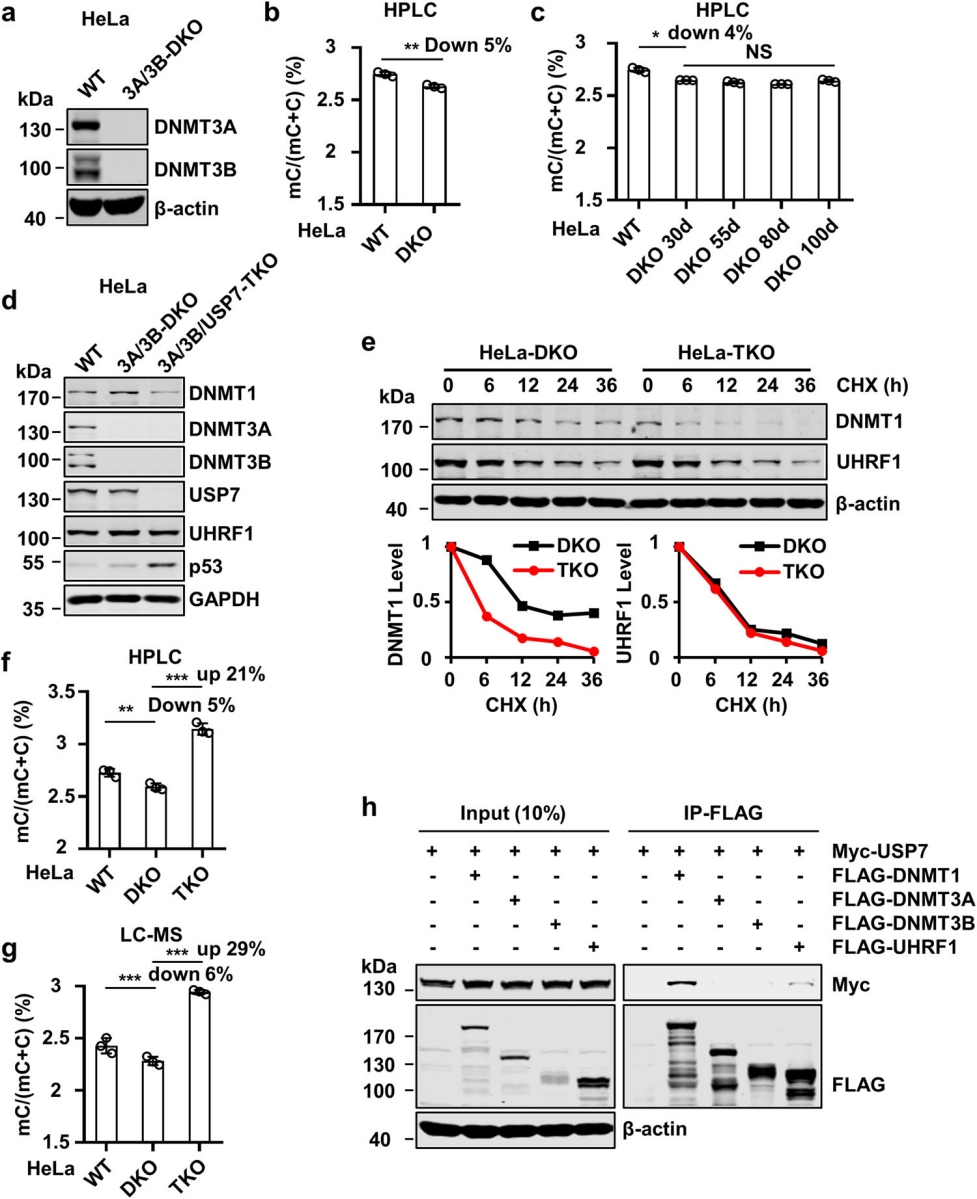
USP7 knockout results in substantially increased DNA methylation in the absence of DNMT3A/3B. **a** WB analysis of control and DNMT3A/3B-DKO HeLa cells. **b** The levels of genomic DNA methylation (mC) in control and DNMT3A/3B-DKO HeLa cells determined by HPLC. **P ≤ 0.01. **c** The levels of genomic DNA methylation (mC) in control and DNMT3A/3B-DKO HeLa cells with progressively increased culture time determined by HPLC. *P ≤ 0.05; NS, not significant. **d** WB analysis showing the levels of key proteins in DNA methylation and p53 in control, DNMT3A/3B-DKO, and DNMT3A/DNMT3B/USP7-TKO HeLa cells. **e** Analysis of DNMT1 and UHRF1 protein stability in DNMT3A/3B-DKO and DNMT3A/DNMT3B/USP7-TKO HeLa cells. The cells were treated with cycloheximide (100 μg/mL) in culture medium for various times as indicated. The WB data were quantified by ImageJ and shown as relative levels to control (0 h) in the lower panel. **f** The levels of genomic DNA methylation (mC) in control, DNMT3A/3B-DKO and DNMT3A/DNMT3B/USP7-TKO HeLa cells determined by HPLC. ***P ≤ 0.001; **P ≤ 0.01. **g** The levels of genomic DNA methylation (mC) in control, DNMT3A/3B-DKO and DNMT3A/DNMT3B/USP7-TKO HeLa cells determined by LC-MS. ***P ≤ 0.001. **h** Co-IP analysis showing the interaction between USP7 and key proteins in DNA methylation. The indicated plasmids were co-transfected into HEK293T cells for 48 h. The cells were harvested, lysed, and enriched with FLAG-M2 beads. The precipitated complexes were subjected to WB analysis.

The progressively increased DNA methylation in USP7-KO cells (Fig. 2g) implies an increased de novo DNA methylation and/or reduced active demethylation by TET proteins. Quantitative measurement of 5hmC by LC-MS revealed insignificant difference between control and USP7-KO cells (data not shown), thus ruling out reduced active demethylation by TETs as the underlying mechanism for increased DNA methylation in USP7-KO cells. To test if DNMT3A and DNMT3B were responsible for increased DNA methylation in USP7-KO cells, we first generated DNMT3A and DNMT3B double knockout (DKO) HeLa cells by CRISPR/Cas9 as reported^48^ (Supplementary Fig. S4a, b). The loss of both DNMT3A and DNMT3B in DKO cells was confirmed by WB analysis (Fig. 4a and Supplementary Fig. S4c) and DNA sequencing (Supplementary Fig. S4d). Consistent with a previous study with DNMT3A/3B-DKO in HCT116 cells^49^, we found that DNMT3A/3B-DKO cells proliferated essentially as the control cells and had a moderate reduction (5%) of global DNA methylation (Fig. 4b). Notably, the global levels of DNA methylation in DNMT3A/3B-DKO cells were stably maintained even after a sustained culture (Fig. 4c), a result again consistent with the previous study with HCT116 cells^49^ but different from mouse and human ES cells in which loss of *Dnmt3a/3b* leads to progressive loss of DNA methylation^50,51^. Thus, DNA methylation can be maintained in a relatively stable level in HeLa cells even in the absence of de novo enzymes DNMT3A/3B.

To test if USP7 negatively regulates DNA methylation by DNMT3A/3B or by DNMT1, we used CRISPR/Cas9 to further knockout USP7 and generated DNMT3A/DNMT3B/USP7 triple knockout (TKO) HeLa cells (Fig. 4d and Supplementary Fig. S4c, d). Such cell lines were viable and had an elevated level of p53 and reduced DNMT1 and insignificant change of UHRF1 proteins (Fig. 4d). Similar to previous USP7-KO cells, TKO cells also exhibited a reduced DNMT1 protein stability (Fig. 4e). Subsequent DNA methylation analysis revealed that, although reduced DNA methylation was observed in the DNMT3A/3B-DKO cells, further deletion of USP7 resulted in a substantial increase of DNA methylation as determined by quantitative HPLC analysis (up by 21%) (Fig. 4f) and LC-MS analysis (up by 29%) (Fig. 4g). These results clearly excluded the possibility that USP7 negatively regulates global DNA methylation by controlling DNMT3A/3B activity. Consistently, co-immunoprecipitation (IP) assay revealed that, while USP7 interacted strongly with DNMT1 and weakly with UHRF1, no interaction with DNMT3A and DNMT3B was detected (Fig. 4h). Taken together, these data indicate that USP7 negatively regulates global DNA methylation through suppression of DNMT1-mediated DNA methylation.

### Loss of USP7 elevates DNA methylation on pre-existing sites and de novo methylation

**Fig. 5:**
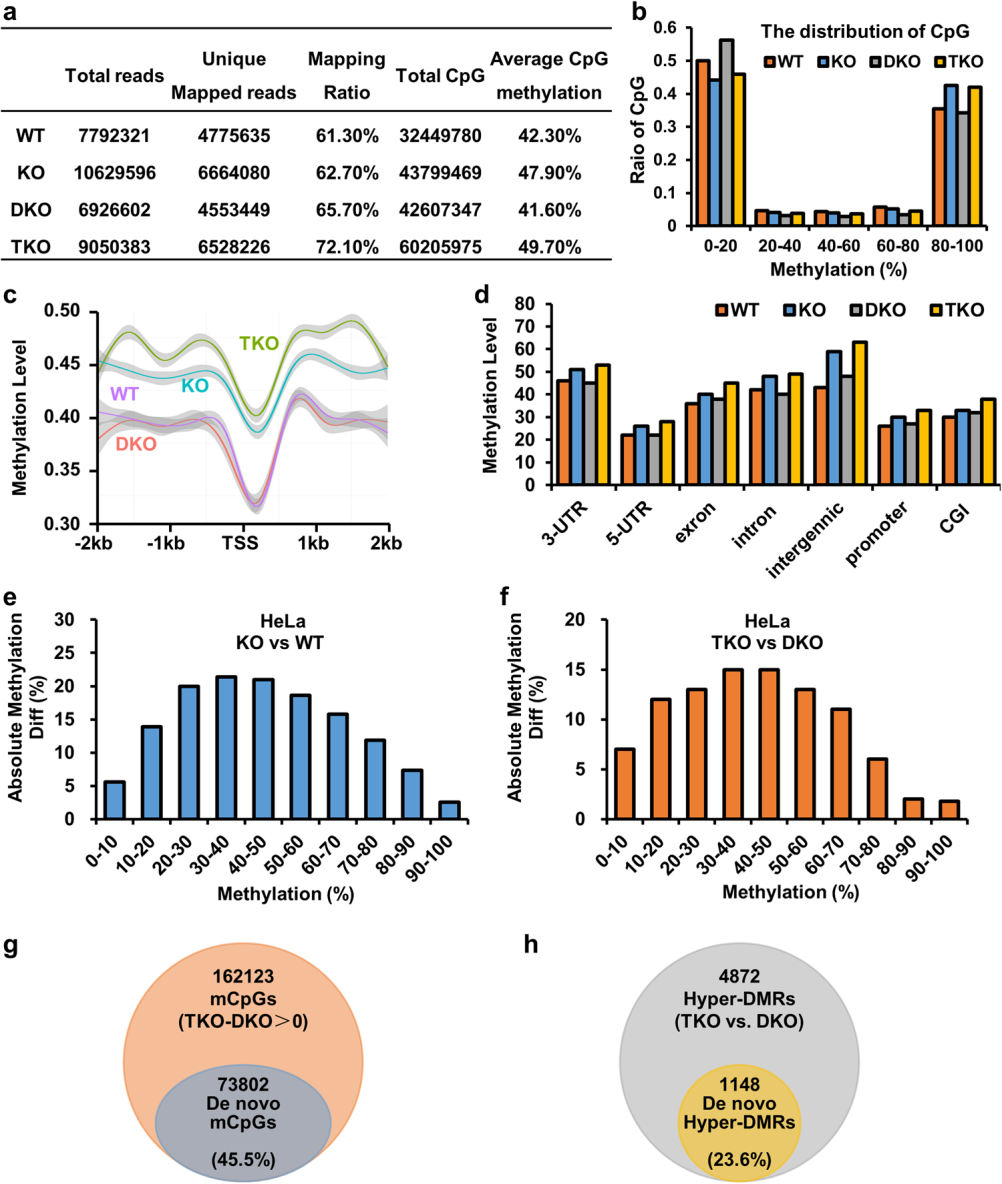
RRBS analysis showing increased DNA methylation in USK7-KO cells. **a** The summary of RRBS data. **b** CpGs with at least 10 reads coverage in WT (control), KO (USP7-KO-1), DKO (DNMT3A/3B-DKO), and TKO (DNMT3A/DNMT3B/USP7-TKO) HeLa cells were plotted based on the percentages of DNA methylation. **c** Average CpG methylation levels in WT, KO, DKO and TKO HeLa cells across the 2-kb upstream (+) and downstream (−) of TSS regions. **d** Methylation differences in various genomic elements of WT, KO, DKO, and TKO HeLa cells in column diagram. **e**, **f** USP7 KO elevates the levels of DNA methylation for pre-existed DNA methylation sites in WT HeLa cells (**e**) or in DKO HeLa cells (**f**). **g**, **h** Venn diagram showing the de novo methylated CpG sites (**g**) or de novo hypermethylated DMRs (**h**) by comparing methylated CpG sites in TKO with DKO cells.

To better characterize the effects of USP7 on DNA methylation, we performed reduced representative bisulfite sequencing (RRBS) analysis^52^ for a genome-wide comparison of DNA methylation in wild-type, USP7-KO-1, DNMT3A/3B-DKO, and DNMT3A/DNMT3B/USP7-TKO HeLa cell lines. As summarized in Fig. 5a, from more than 4 million of unique reads for each RRBS data, the average level of CpG methylation was found to be 47.90% in USP7-KO cells and 42.30% in control HeLa, indicating a 13.2% increase of DNA methylation in USP7-KO-1 cells. While average CpG methylation was reduced slightly to 41.60% in DNMT3A/3B-DKO cells, it increased to 49.70% in DNMT3A/DNMT3B/USP7-TKO cells, representing a remarkable 19.5% increase of DNA methylation upon USP7 KO. As shown in Fig. 5b, the patterns of DNA methylation in all four cell types show a typical biphasic distribution, with a shift to increased DNA methylation in both USP7-KO and DNMT3A/DNMT3B/USP7-TKO cells. When CpG sites were aligned by their relative distance to transcriptional start sites, we observed that USP7 KO resulted in increased DNA methylation across the 2-kb upstream (+) and downstream (−) of TSS regions (Fig. 5c). Further analysis of DNA methylation based on genomic elements also revealed an across-the-board increase of DNA methylation in USP7-KO cells and DNMT3A/DNMT3B/USP7-TKO cells (Fig. 5d and Supplementary Fig. S5). Indeed, when methylated CpG sites in control or DNMT3A/3B-DKO HeLa cells were separated into ten quantiles according to their levels of DNA methylation, increased levels of DNA methylation were observed for each quantile in comparison of USP7-KO to control cells (Fig. 5e) or DNMT3A/DNMT3B/USP7-TKO cells to DNMT3A/3B-DKO (Fig. 5f). Importantly, comparison of differentially methylated CpG sites in DKO and TKO cells revealed a substantial fraction (45.5%) of de novo (new) methylated CpG sites, among all sites with increased levels of methylation (Fig. 5g). By arbitrarily defining differentially methylated regions (DMRs) as regions equal or more than 1 kb with at least a 25% increase of DNA methylation, comparison of DKO and TKO RRBS data revealed 4872 hypermethylated DMRs in TKO cells, and among them 1148 were de novo hypermethylated DMRs (Fig. 5h). Altogether, deletion of USP7 in DNMT3A/3B-DKO cells not only led to elevated levels of DNA methylation in pre-existing methylated CpG sites, it also resulted in a substantial fraction of de novo methylation.

### Loss of USP7 enhances DNMT1 association with replication sites

**Fig. 6:**
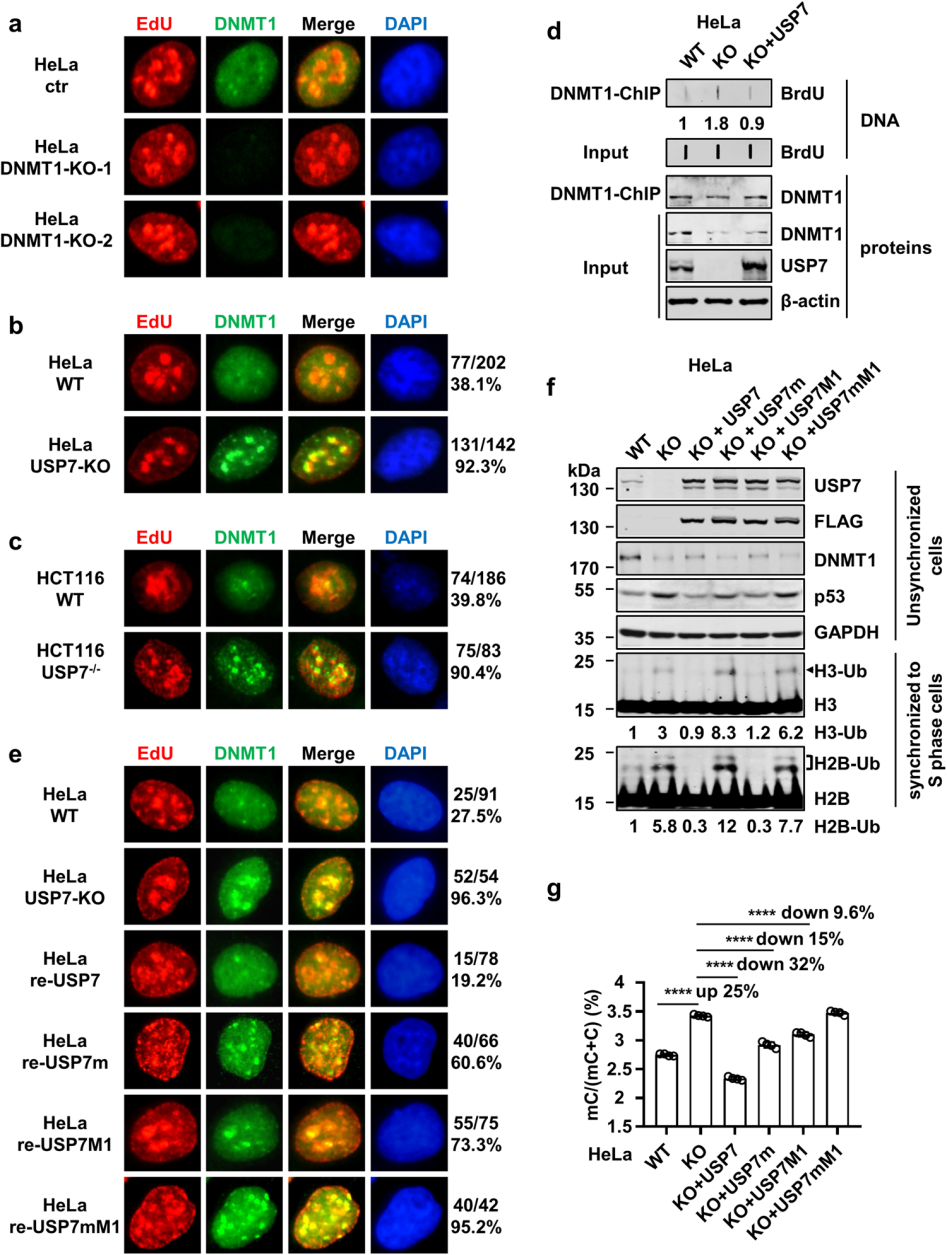
Both physical interaction with DNMT1 and deubiquitinase activity are required for USP7 to inhibit DNMT1 replication fork association and DNA methylation in USP7-KO cells. **a** Validation of DNMT1 antibody for detection of endogenous DNMT1 by immunostaining. Essentially no DNMT1 signal was detected in DNMT1-KO cells by this anti-DNMT1 antibody. **b**, **c** Representative images showing a markedly increased level of DNMT1 protein in the EdU positive foci, which corresponded to replicating heterochromatin regions in USP7-KO HeLa cells (**b**) and USP7−/− HCT116 cells (**c**). Also indicated are the ratios and numbers of counted EdU foci-positive cells with co-localized DNMT1 foci. **d** ChIP-slot blot experiments showing enhanced association of DNMT1 with BrdU-labeled DNA in USP7-KO HeLa cells. Control, USP7-KO, and FLAG-USP7-expressing USP7-KO HeLa cells were labeled with BrdU for 20 min before subjected to ChIP with DNMT1. The obtained DNA samples were than analyzed by slot blot using anti-BrdU antibody and the relative BrdU signals were quantified. Also shown were WB data for USP7 and DNMT1 before and after immunoprecipitation. **e** Representative images showing the effect of various ectopically expressed USP7 mutants on DNMT1 foci formation in USP7-KO HeLa cells. Also indicated are the ratios and numbers of counted EdU foci-positive cells with co-localized DNMT1 foci. **f** WB analysis of USP7-KO HeLa cells that stably re-expressed wild-type or various USP7 mutants. For upper panel, the unsynchronized cells were used. For detecting histone ubiquitination by WB, core histones were prepared by acid extraction from cells synchronized to S phase. **g** HPLC analysis of the levels of genomic DNA methylation (mC) in control, USP7-KO, and stably re-expressing wild-type or various USP7 mutants USP7-KO HeLa cell lines. ****P ≤ 0.0001.

As increased histone ubiquitination is observed in USP7 KO cells, we next examined if loss of USP7 results in an increase of DNMT1 recruitment in S phase of cell cycle. It is well established that DNMT1 exhibits a characteristic co-localization with pericentric heterochromatin foci in mid to late S phase of the cell cycle during which the pericentric heterochromatin DNA is replicated^53^. To examine whether USP7 controls the recruitment of DNMT1 to pericentric heterochromatin in S phase of the cell cycle, we first tested the specificity of anti-DNMT1 antibody for detection of endogenous DNMT1 by immunostaining of DNMT1-KO cells generated by CRISPR-Cas9 method. As shown in Fig. 6a (also see Supplementary Fig. S6a, b), no immunostaining signal was detected by this antibody in DNMT1-KO cells, thus validating the specificity of this antibody. We then labeled both control and USP7-KO HeLa cells with EdU (5-Ethynyl-2′-deoxyuridine) for 30 min to mark S-phase cells and then carried out immunofluorescent staining for both EdU and DNMT1. We specifically compared the DNMT1 staining patterns in the late S phase cells marked by strong EdU foci staining. As shown in Fig. 6b, in control HeLa cells ~38.1% strong EdU foci cells had relatively weak but co-localized DNMT1 foci, consistent with an enrichment of DNMT1 in late replicating pericentric heterochromatin. Notably, in USP7-KO cells 92.3% strong EdU foci cells had stronger and co-localized DNMT1 staining foci (Fig. 6b and Supplementary Fig. S6c). Similarly, USP7^−/−^ HCT116 cells also showed stronger and clear DNMT1 foci in late S-phase cells (Fig. 6c and Supplementary Fig. S6d). Thus, consistent with an increased DNA methylation phenotype, loss of USP7 apparently enhanced DNMT1 association with replicating DNA, a result consistent with a previous study^44^.

To validate that loss of USP7 indeed enhances DNMT1 recruitment to replicating DNA, we measured the amount of DNMT1 associated with replicating DNA. We first labeled replicating DNA in control, USP7-KO, and FLAG-USP7-expressing USP7-KO HeLa cells with BrdU for 20 min. The cells were then treated with formaldehyde and subjected to chromatin immunoprecipitation by using anti-DNMT1 antibody. Immunoprecipitated DNMT1 was checked by WB to ensure the similar amount of DNMT1 was obtained from each sample (Fig. 6d). The DNMT1-associated DNA was then recovered and the level of BrdU-labeled DNA was determined by slot blot using anti-BrdU antibody. In three experiments we found that USP7-KO led to an average of 1.8-fold increased association of DNMT1 with BrdU labeled DNA, despite a reduced global level of DNMT1 proteins in the USP7-KO cells (Fig. 6d). Furthermore, re-expression of USP7 in USP7-KO cells reversed this effect, indicating that the increased association of DNMT1 with replicating DNA is due to loss of USP7.

### Both physical interaction with DNMT1 and deubiquitinase activity are required for USP7 to suppress DNMT1 replication fork association and DNA methylation

A previous study^13^ showed a DNMT1-dependent deubiquitination of H3. In addition, our co-IP assay showed that USP7 interacts strongly with DNMT1 and weakly with UHRF1 (Fig. 4h), raising the possibility that, while DNMT1 itself is recruited to replicating DNA at least in part by binding ubiquitinated histones, DNMT1 may recruit USP7 by physical interaction, which in turn deubiquitinates histones to attenuate DNMT1’s association with replicated DNA. To test this hypothesis, we took advantage of bigger and brighter DNMT1 foci in S phase of USP7-KO HeLa cells. We ectopically expressed wild-type and different USP7 mutants in USP7-KO HeLa cells and assessed their ability to inhibit the formation of big DNMT1 foci in S phase. The results in Fig. 6e (also see Supplementary Figs. S7 and S8) show that while strong and EdU-co-localized DNMT1 foci was observed in 96.3% USP7-KO cells, ectopic expression of wild-type USP7 markedly reduced the intensity of DNMT1 foci and the rate of cells with EdU and DNMT1 co-localization to 19.2%, demonstrating that the loss of USP7 counts for enhanced DNMT1 recruitment in USP7-KO cells. However, re-expression of either the USP7m mutant defective in deubiquitinase activity or the USP7M1 mutant defective in DNMT1 interaction only moderately reduced DNMT1 foci intensity and rate of co-localization with EdU foci (Fig. 6e). On the other hand, the compound USP7 mutant (USP7mM1) defective in both deubiquitinase activity and DNMT1 interaction were essentially inactive in suppressing DNMT1 foci intensity and co-localization with EdU foci. These results indicate that suppression of DNMT1 replication site association by USP7 requires both its physical interaction with DNMT1 and deubiquitinase activity.

Consistent with previous report that USP7 is primarily a nuclear protein and co-localizes with DNMT1 or PCNA in the late S phase^42,45^, we also observed that USP7 forms foci and co-localizes with EdU foci in late S phase (Supplementary Fig. S8c). There is no significant change of USP7 protein level throughout the cell cycle (Supplementary Fig. S8d).

To investigate if inhibition of DNMT1 recruitment by USP7 correlates with its ability to inhibit DNA methylation, we introduced wild-type or mutant USP7 into USP7-KO HeLa cells by transfection and obtained pools of stable cells with comparable stable expression of exogenous FLAG-USP7 (Fig. 6f). Note a reduced level of p53 in both USP7- and USP7M1-expressing KO cells but not in USP7m- and USP7mM1-expressing KO cells, consistent with a role of USP7 deubiquitinase activity in the control of p53 protein stability^33,34^. Also consistent with a role in stability of DNMT1 through deubiquitination, re-expression of both wild-type USP7 and USP7M1 partially restored the level of DNMT1 in USP7-KO cells (Fig. 6f). As histone H3 ubiquitination is hardly detected outside of S phase, we also synchronized the cells to S phase and examined the status of H3 and H2B ubiquitination by WB analysis. Re-expression of wild-type USP7 and USP7M1 markedly downregulated the level of ubiquitinated H3 and H2B in S phase, suggesting that USP7 can deubiquitinate bulk H3 and H2B in a DNMT1 interaction-independent manner. Subsequent quantitative analysis of DNA methylation revealed that re-expression of wild-type USP7 completely reversed the elevated DNA methylation phenotype in USP7-KO cells (from 3.43 to 2.34%, down by 32%) (Fig. 6g). In fact, the level of DNA methylation in FLAG-USP7-expressing USP7-KO cells was even lower than that of control HeLa cells (2.34% mC vs. 2.75% mC), presumably due to a higher than the wild-type level of USP7 proteins in the re-expressing USP7 cells. Expression of USP7m and USP7M1 reduced the level of DNA methylation in USP7-KO HeLa cells to 2.92% and 3.10%, down by 15% and 9.6%, respectively. Notably, expression of the compound USP7mM1 mutant did not reduce DNA methylation at all. This quantitative analysis of DNA methylation revealed a nice correlation between the inhibition of DNA methylation and inhibition of DNMT1 recruitment by USP7. Taken together, our data indicate that USP7 inhibits DNMT1 replication fork association and global DNA methylation via its deubiquitinase activity and physical interaction with DNMT1.

## Discussion

In this study, we present evidence that USP7 negatively regulates global DNA methylation by suppressing DNMT1 recruitment to replication fork. This negative regulation is required to prevent excessive DNA methylation by DNMT1, thus promoting DNA methylation homeostasis and stable inheritance of DNA methylation.

Previous studies have identified USP7 as a major DNMT1 and UHRF1-interacting protein. USP7 was shown to stabilizing DNMT1^38,40,42^ and UHRF1^39,41^ by protein deubiquitination. However, Yarychkivska et al. recently reported that knockout of USP7 in mouse embryonic fibroblasts (MEFs) and knockdown of USP7 in H1299 cells had no significant effect on the global levels of DNMT1 protein^45^. We showed that loss of USP7 reduced the protein stability of both DNMT1 and UHRF1 in HeLa. In HCT116 cells, we found that loss of USP7 affected DNMT1 protein stability more than that of UHRF1. Thus. USP7 appears to regulate DNMT1 and UHRF1 protein stability in a cell-type dependent manner. Despite a reduced protein stability, we found that loss of USP7 did not significantly affect the overall levels of UHRF1 and DNMT1 in HeLa cells, most likely due to an increased expression of DNMT1 and UHRF1 in USP7-KO cells (Supplementary Fig. S2e). Thus, loss of USP7 can variably affect the global level of DNMT1 and UHRF1, despite a more general role for USP7 in stabilizing both proteins.

Given a role for USP7 in stabilizing UHRF1 and DNMT1, the major and most surprising finding in our study is that USP7 knockout results in a significant increase of global DNA methylation in HeLa, HCT116, and mouse embryos. Du et al. and Yarychkivska et al. have investigated the effect of USP7 knockout and/or knockdown on global DNA methylation and concluded that knockout or knockdown of USP7 did not significantly alter the global level of DNA methylation^38,45^. Felle et al. showed that USP7 stimulated both maintenance and de novo methylation activity of DNMT1 in vitro^39^, and knockdown of USP7 reduced DNA methylation in three silenced genes, although whether USP7 knockdown affected global DNA methylation was not analyzed. Yamaguchi et al. reported that depletion of USP7 impaired DNA methylation on replicating DNA in Xenopus egg extracts^44^. A caveat in this experiment is that, given the observed interaction between USP7 and DNMT1, depletion of USP7 would also reduce DNMT1 and thus impaired DNA methylation. The discrepancies with regard to the role of USP7 in DNA methylation among literature and between previous and the current studies could be explained in part by the nature of DNA maintenance methylation and differences in experimental approaches. In previous studies, the effect on global DNA methylation was measured with cells upon acute KO or knockdown of USP7. As these cells had undergone only a few (~2–4) cell division cycles, these experiments most likely were not sensitive enough to detect elevated but weak de novo activity from DNMT1. Consistent with this idea, we failed to detect any significant increase in global DNA methylation upon acute knockdown of USP7 by siRNA (Supplementary Fig. S2h, i). We were able to uncover a critical but suppressive role for USP7 in DNA methylation because USP7-KO cell lines used in our study were derived from single USP7-KO cells. These cells had undergone many more cell divisions than acute knockout or knockdown cells used in previous studies. In addition, it is formally possible that USP7 stimulates DNA methylation at a fraction of genomic regions, although overall it suppresses global DNA methylation. This could explain why reduced DNA methylation was observed by Felle et al. upon knockdown of USP7^39^.

By four different assays (HPLC, LC-MS, bisulfite sequencing of LINE1, and RRBS) we showed that USP7-KO HeLa cells had a substantially increased DNA methylation (Figs. 2, 5). The negative effect of USP7 on DNA methylation was confirmed by deleting USP7 in HCT116 cells and mouse embryos (Fig. 3). Importantly, this negative regulation on DNA methylation was observed in DNMT3A/3B-DKO HeLa cells (Figs. 4, 5), thus demonstrating unambiguously that USP7 negatively regulates DNMT1-mediated DNA methylation. Consistent with a net increase of global DNA methylation, RRBS analysis revealed increased DNA methylation not only for pre-existing methylation sites but also a substantial fraction of de novo methylation sites in DNMT3A/DNMT3B/USP7-TKO HeLa cells (Fig. 5). As a marked increase of DNA methylation (up to 29%) was observed in the DNMT3A/DNMT3B/USP7-TKO cells, our study revealed that DNMT1 exhibits a substantial de novo methyltransferase activity in the absence of USP7. In this regard, we like to point out that DNMT1 must have certain level of de novo activity even in the presence of USP7, because DNMT3A/3B-DKO HeLa cells are able to stably maintain DNA methylation (Fig. 4c) and because maintenance methylation by DNMT1 cannot be 100% in efficiency (95–96% in vitro)^54^. DNMT1 has been shown to exhibit de novo activity in vitro^7,8^ and in mouse ES cells^10^. In fact, a recent study demonstrated that Stella is required for protecting mouse oocytes from excessive DNA methylation by inhibiting DNMT1 de novo activity^55^. As DNMT3A/3BUSP7-TKO HeLa cells showed progressively increased DNA methylation, USP7 is clearly required for suppressing DNMT1 de novo activity. Our study thereby reveals for the first time that USP7 safeguards somatic genome from excessive DNA methylation by suppressing DNMT1, especially its de novo activity.

Consistent with reported deubiquitinase activity for H2B^37^ and H3^44^, we observed significantly elevated levels of ubiquitinated H2B and H3, especially in S phase, in USP7 KO cells. UHRF1 was previously shown to ubiquitinate H3 at multiple sites^13,14,31^. We found that UHRF1 ubiquitinates not only H3 but also H2B and is responsible for an increase of ubiquitinated H2B and ubiquitinated H3 in S phase of the cell cycle (Fig. 1). Our data also suggest that UHRF1 ubiquitinates H2B at site(s) other than K120 and the exact site(s) of ubiquitination remains to be determined. Nevertheless, a functional relevance of ubiquitinated H2B in DNA methylation is supported by our observation that DNMT1 binds not only ubiquitinated H3 but also ubiquitinated H2B through its UIM domain (Fig. 1g). Our data indicate that UHRF1 and USP7 oppositely control dynamic ubiquitination of H3 and H2B. It is noteworthy that the overall level of H2B ubiquitination in S phase is higher than that of H3 ubiquitination, suggesting it may play an important role in DNMT1 recruitment.

Consistent with a role of histone ubiquitination in DNMT1 recruitment, we observed that USP7 knockout led to a substantially elevated association of DNMT1 with replication sites. An elevated DNMT1 association with replicating heterochromatin with knockdown of USP7 was also observed in a previous study^44^. We show that ectopic expression of wild-type USP7 can completely reverse this phenotype, and this ability depends on its deubiquitinase activity and interaction with DNMT1. We believe that the enhanced DNMT1 association with replication sites most likely accounts for increased DNA methylation in USP7 KO cells.

**Fig. 7:**
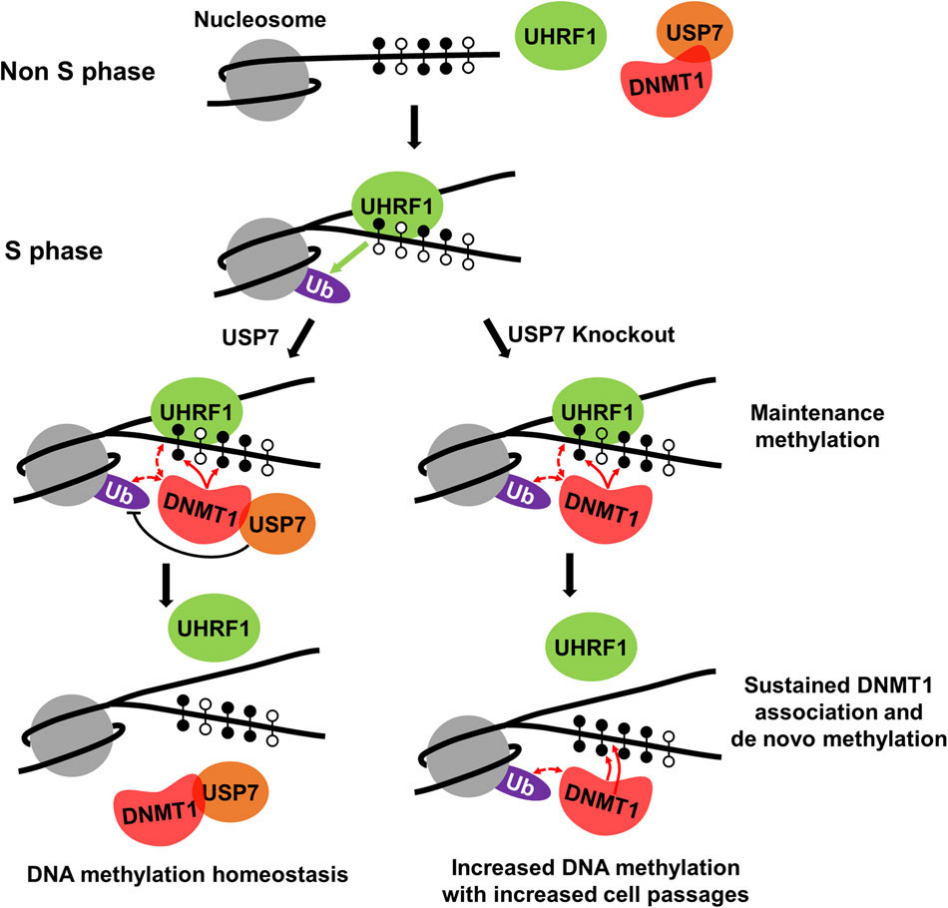
Working model illustrating how USP7 negatively regulates global DNA methylation. The diagrams illustrate how USP7 suppresses excessive DNA methylation by DNMT1 to promote DNA methylation homeostasis. In S phase cells, UHRF1 binds to replicating fork by binding of hemi-methylated DNA. Binding of hemi-methylated DNA is likely to cause UHRF1 conformational change and enhances its ability to catalyze H3 and H2B ubiquitination. DNMT1 is then recruited by binding both UHRF1 and ubiquitinated H3 and/or H2B and exerts its maintenance methylation activity. USP7 is also recruited to replication fork, at least in part through its interaction with DNMT1. The recruitment of USP7 leads to deubiquitination of H3 and H2B at replication fork. H3 and H2B deubiquitination, together with dissociation of UHRF1 from replicating fork due to conversion of hemi-methylated to fully methylated CpGs by DNMT1, leads to dissociation of DNMT1 from DNA. However, in the absence of USP7 the ubiquitinated H3/H2B at replication fork is not deubiquitinated in time, leading to more stable and extended association of DNMT1, which in turn leads to increased de novo DNA methylation by DNMT1. The de novo methylated sites in one cell cycle will be maintained in next round of cell division and results in progressively elevated DNA methylation upon increased cell passages.

Taken together the previously published data and data presented here, we propose a working model how USP7 negatively controls DNA methylation by DNMT1 and promotes DNA methylation homeostasis (Fig. 7). When cell enters S phase, DNA replication generates hemi-methylated CpGs at the replication sites. UHRF1 binds hemi-methylated CpGs, which induces UHRF1 conformational change and allows UHRF1 to efficiently ubiquitinate histones H3 and H2B^31,56,57^. Binding of H3K9 methylated H3 and/or methylated DNA ligase 1 also help localize UHRF1 to replication sites^23,24,29^. DNMT1 is then recruited to replicating fork by binding of ubiquitinated histones and interaction with UHRF1 and converts hemi-methylated CpGs to fully methylated DNA to fulfill its maintenance methylation function^13,14^. Under the normal condition, the recruitment of DNMT1 can also lead to recruitment of USP7, which deubiquitinates H3 and H2B. Histone deubiqutination by USP7 would dissociate DNMT1 from replicated sites, thus reducing the chance of de novo DNA methylation by DNMT1. In the absence of USP7, UHRF1-catalyzed H2B/H3 ubiquitination accumulates on replication sites and results in enhanced and prolonged DNMT1 association. The elevated and prolonged DNMT1 association presumably not only enhances DNMT1 maintenance but also do novo methyltransferase activities. As the resulting de novo methylated CpGs will be maintained in next cell division, a small increase in DNMT1 de novo activity in principle can be accumulated and propagated with increased cell divisions and eventually lead to a substantial increase of global DNA methylation. Thus, USP7 not only plays a role in stabilizing DNMT1 and UHRF1, but also serves to suppress excessive DNA methylation by DNMT1.

## Materials and methods

### Cell culture

Human embryonic kidney 293T cell line (HEK293T), human cervical cancer cell line (HeLa), and mouse embryo fibroblast cell line (NIH3T3) were maintained in DMEM medium (Gibco) containing 10% fetal calf serum (Gemini), 100 U/mL penicillin and 100 μg/mL streptomycin (Gibco). Human colon cancer cells (HCT116) was maintained in McCoy’s 5A medium (Gibco) containing 10% fetal calf serum, 100 U/mL penicillin, and 100 μg/mL streptomycin.

### Antibodies and constructs

The following primary antibodies were used in this study: USP7 (GeneTex, #GTX25894; 1:1000 for western blotting), USP7 (Zen Bioscience, #200333; 1:1000 for western blotting), p53 (Santa Cruz, #sc-126; 1:500 for western blotting), DNMT1 (Santa Cruz, #sc-20701; 1:500 for western blotting and 1:100 for immunostaining), DNMT1 (our laboratory, #2B4; 1:500 for western blotting and immunostaining), UHRF1 (Proteintech, #21402-1-AP; 1:1000 for western blotting and immunostaining), DNMT3A (Santa Cruz, #sc-20703; 1:2000 for western blotting), DNMT3B (GeneTex, #GTX129127; 1:1000 for western blotting), β-actin (HUABIO, #M1210-2; 1:5000 for western blotting), GAPDH (Abmart, #M20006L; 1:5000 for western blotting), HA (Abmart, #M20003; 1:5000 for western blotting), FLAG (Sigma, #F1804; 1:5000 for western blotting and immunostaining), Myc (Abmart, #M20002, 1:5000 for western blotting), histone H3 (Abcam, ab1791; 1:5000 for western blotting), histone H2B (Proteintech, #15857-1-AP; 1:500 for western blotting), H3S10P (Epitomics, #1173-1; 1:10,000 for western blotting), 5-methylcytidine (Eurogentec, #BI-MECY-0500; 1:5000 for immunostaining), BrdU (Sigma, #B8434; 1:10,000 for western blotting). The following secondary antibody were used: Alexa Fluor 680 goat anti-rabbit IgG (Jackson ImmunoResearch, #111-625-144; 1:10,000 for western blotting); Alexa Fluor 790 goat anti-mouse IgG (Jackson ImmunoResearch, #115-655-146; 1:10,000 for western blotting); Alexa Fluor 594 goat anti-rabbit IgG (Jackson ImmunoResearch, #111-585-003; 1:500 for immunostaining); Alexa Fluor 488 goat anti-mouse IgG (Jackson ImmunoResearch, #115-545-003; 1:500 for immunostaining).

UHRF1, DNMT3A, DNMT3B, and DNMT1 cDNAs were subcloned into the FLAG-tagged vector (pcDNA3.1). USP7 cDNA was subcloned into pPy-CAGIP vector and all mutants were generated by site-directed mutagenesis and confirmed by sequencing. H2A, H2B, H3, and H4 cDNAs were subcloned into the FLAG-tagged vector (p3×FLAG). DNMT1-N (329-642 aa) and DNMT1-N-ΔUIM (Δ392-406 aa) DNA sequences were cloned into the PGEX4T-1 vector.

### RNAi

For siRNA transfection, cells were transfected twice at 24-h intervals with the indicated siRNA using Lipofectamine 2000 (Invitrogen, Waltham, MA) following the manufacturer’s instructions. The sequence of siRNA against human UHRF1 were GGGUGGUGCGCAAUGUCAAGG. The sequence of siRNA against human USP7 were GCAUAGUGAUAAACCUGUAGG.

### CRISPR/Cas9 system

The USP7-KO, DNMT3A/3B-DKO, and DNMT3A/DNMT3B/USP7-TKO HeLa cell lines were obtained by CRISPR system essentially as described^58^ with the guide RNAs listed below. GTACATGATGCCAACCGAGG for USP7; GCTACCACGCCTGAGCCCGT for DNMT3A; AGACTCGATCCTCGTCAACG for DNMT3B. The knockout of *Usp7* mice embryos was obtained essentially as described^47^ with some modification. In brief, two 20-nt guide sequence 5′ to a NGG PAM (Usp7-1: TTGCCTCGGAGCGCCAAC and Usp7-2: TCCTACGCTTTTTTGGTG) were selected to synthesize sgRNA templates. In vitro synthesized Cas9 mRNA and sgRNAs were co-injected into the cytoplasm of one-cell-stage mice embryos. The control and injected embryos were cultured in M2 medium (Gibco) in vitro for 3 days to allow embryos to develop to morula stage. The embryos were then collected for genotyping and DNA methylation analysis by bisulfite sequencing.

### Immunoprecipitation assay

For co-IP of exogenous proteins, the indicated plasmid(s) were transfected into HEK293T cells. The cells were collected 48 h after transfection and lysed in IP Lysis buffer (25 mM Tris-HCl, pH 8.0, 150 mM NaCl, 1% NP-40, 2 mM EDTA, 1× protease inhibitor cocktail, 1 mM DTT). The lysates were cleared by centrifugation at 12,000 rpm for 20 min at 4 °C. The supernatant was directly incubated with anti-FLAG M2-affinity beads (Bimake) for 3 h at 4 °C. After extensive washing with lysis buffer, complexes were boiled in 1×SDS loading buffer and analyzed by SDS-PAGE.

For denature immunoprecipitation assay for ubiquitinated histones was performed as described^13^.

### Histone acid extraction

Preparation of core histones by acid extraction was performed as described^13^. The cells were lysed in 1×PBS with 0.5% Triton X-100 and protease inhibitor at 4 °C for 20 min. The lysates were cleared by centrifugation at 12,000 rpm at 4 °C for 10 min and the pellets were rinsed once in the lysis buffer. The histones were then extracted in 0.2 N HCl at 4 °C for 30 min. The lysates were centrifuged at 4 °C for 10 min at 12,000 rpm, and the supernatants were collected and adjusted to pH 7.5 with 2 M Tris.

### In vitro deubiquitinase enzymatic assay

To purify FLAG-tagged USP7 or mutant proteins from mammalian cells, the HEK293T cells were transfected with plasmids encoding FLAG-USP7 or enzymatic mutant USP7m for 48 h. The cells were collected and lysed in high salt Lysis buffer (25 mM Tris-HCl, pH 8.0, 500 mM NaCl, 1% Triton X-100, 2 mM EDTA, 1× protease inhibitor cocktail, 1 mM DTT). These FLAG-tagged proteins were then captured with anti-FLAG M2-affinity beads and eluted with FLAG-peptide elution buffer (100 μg/mL FLAG-peptides, 50 mM Tris-HCl, pH 8.0, 10% glycerol, 1 mM EDTA, 1× protease inhibitor cocktail, 1 mM DTT). For preparation of ubiquitinated histone substrates, HEK293T cells were transfected with UHRF1 expression plasmids for 48 h and synchronized to the G1/S boundary by aphidicolin treatment for 18 h, followed by release from arrest for 4 h. The core histones including ubiquitinated histones were prepared by acid extraction. For in vitro deubiquitinase enzymatic assay, ~2 μg core histones and 0.5 μg FLAG-USP7 or FLAG-USP7m were incubated in 20 μL reactions (50 mM Tris-HCl, pH 8.0, 10% glycerol, 1 mM EDTA, 1× protease inhibitor cocktail, 1 mM DTT) at 37 °C for 1 h, followed by SDS-PAGE and WB analysis.

### Purification of GST-tagged proteins

For purification of recombinant protein, pGEX-4T-1-GST-DNMT1-N or DNMT1-N (ΔUIM) plasmids were transformed into *E. coli* BL21. The bacteria were cultured in LB medium with 100 μg/mL ampicillin at 37 °C until reaching on optical density of 0.6–0.7 at 600 nm. The expression of recombinant proteins was induced by 0.1 mM IPTG (Isopropyl β-D-1-thiogalactopyranoside) for 2 h at 24 °C. The cells were pelleted and lysed by sonication in 1×PBS buffer containing 10% (W/V) glycerol, 1× protease inhibitor cocktail, and 1 mM DTT. After the debris was removed by centrifugation at 9500 rpm for 15 min, the supernatants were incubated with Glutathione Sepharose (GenScript) for at least 3 h at 4 °C with gentle rotation. The beads were washed five times with cold PBS buffer containing 0.05% Triton X-100 and then eluted with elution buffer (50 mM Tris-HCl, pH 7.5, 10 mM Glutathione, 10% glycerol, 1× protease inhibitor cocktail, and 1 mM DTT).

### GST pull-down assay

In the GST pull-down system, the GST-tagged proteins captured on the Glutathione Sepharose beads and the core histones prepared from FLAG-H3 or FLAG-H2B and UHRF1 transfected HEK293T cells by acid extraction were incubated in binding buffer (25 mM Tris-HCl pH 8.0, 150 mM NaCl, 1% NP-40, 2 mM EDTA, 1× protease inhibitor cocktail, and 1 mM DTT) at 4 °C for 3 h on a rotator. The beads were washed three times with binding buffer, and the bound core histones were detected by WB analysis.

### Immunofluorescence staining assay

Cells grown on slides were washed with 1×PBS and fixed with 4% paraformaldehyde for 20 min at 4 °C. After fixation, the cells were permeabilized with 1% Triton X-100 in 1×PBS for 20 min at 4 °C. The cells were then blocked with 5% bovine serum albumin (BSA) in 1×PBS for 1 h at 37 °C and incubated with primary antibodies as indicated overnight at 4 °C. After washing three times with 1×PBS, the cells were incubated with Alexa Fluor 594 goat anti-rabbit IgG and Alexa Fluor 488 goat anti-mouse IgG at 37 °C for 1 h. Finally, the nuclei were stained by Hoechst 33342 (Sigma). Followed by washing three times with 1×PBS, the slides were visualized on a Leica DM4000 Microsystems. The fluorescent intensity spectrum of indicated single cell was analyzed by Volocity 6.3.

### EdU staining assay

The EdU staining assays was performed according to the Cell-Light TM EdU Fluorescent Detection Kit (RiBoBio, #C10310) with a slight modification. In brief, cells grown on 48 wells were labeled with 20 μM EdU 5(-ethynyl-2′-deoxyuridine) for ~30 min at 37 °C, washed with 1×PBS twice and fixed with 4% paraformaldehyde for 20 min at 4 °C before neutralization with glycine (2 mg/mL). Then the cells were permeabilized, blocked and incubated with antibodies as described above. Finally, the EdU were stained by Apollo reaction buffer at 37 °C for 30 min. Slides were washed by methanol once and PBS twice before fluorescent imaging.

### ChIP-slot blot

The control, USP7-KO and FLAG-USP7-expressing USP7-KO HeLa cells were synchronized to S phase and labeled with BrdU (Sigma) for 20 min. The cells were cross-linked with 1% formaldehyde for 15 min before neutralization with 0.125 M glycine. The cells were lysed by sonication in ChIP Lysis buffer (25 mM Tris-HCl, pH 8.0, 0.1% SDS, 1 mM EDTA, 1× protease inhibitor cocktail), and soluble cell extracts were recovered after centrifugation at 12,000 rpm and 4 °C for 20 min. The supernatant was incubated with DNMT1 antibody overnight at 4 °C on a rotator. Chromatin-antibody complexes were isolated with 20 μL of Protein G beads blocked by sperm DNA and bovine serum albumin. After extensive washing, protein/DNA complexes were eluted from the beads in Elution buffer (50 mM Tris-HCl, pH 8.0, 1% SDS, 10 mM EDTA) at 65 °C overnight. Immunoprecipitated DNA was purified by phenol/chloroform extraction, and analyzed by slot blot using BrdU antibody.

### HPLC analysis of 5mC

To prepare genomic DNA, cells were resuspended with cell lysis buffer (10 mM Tris-HCl, pH 7.5, 10 mM EDTA, 10 mM NaCl, 0.5% sarcosyl, 0.1 mg/mL RNase (CWBIO)) and incubated at 37 °C overnight. Then, protease K (Merck) was added to a final concentration of 0.2 mg/mL and incubated at 65 °C for 24 h. Genomic DNA was extracted by phenol/chloroform and ethanol precipitated. The DNA was dissolve in ddH_2_O. To hydrolyzs the genomic DNA, 25 μg of denatured genomic DNA were incubated overnight in 133 μL of the hydrolysis solution (40 mM NaAc pH 5.3, 1 mM ZnSO_4_, 1.5 U/mL nuclease P1 (Wako, #145-08221)) at 37 °C overnight and 20 U CIP (NEB, #M0290s) was then added and incubated for additional 4 h at 37 °C. Fifty microliters of hydrolyzate were then analyzed using a HPLC system (Agilent Technologies, 1100 Series) equipped with an Agilent Eclipse XDB-C18 column (5 μm, 4.6 × 250 mm, Agilent Technologies Inc.). dCMP and 5-me-dCMP were detected by UV-detector at 280-nm wavelength.

### LC-MS analysis of 5mC

Genomic DNA was extracted from the cultured cells as above. The extracted DNA (5 μg) was digested to nucleosides with 1.0 U DNase I, 2.0 U calf intestinal phosphatase, and 0.005 U snake venom phosphodiesterase I at 37 °C overnight and then subjected to LC-MS analysis as described^59^.

### Bisulfite sequencing

Bisulfite conversion was performed using the EZ DNA Methylation-Gold^TM^ Kit (ZYMO Research) according to the Instruction manual. Bisulfite converted DNA was used in PCR amplification by TaKaRa Ex Taq HS (Takara). The PCR products were purified by gel extraction for TA clone and sequencing. The primers for PCR amplification are listed below:

5′-GAGATTATATTTTATATTTGGTTTAGAGGG-3′ and 5′-AACTATAATAAACTCCACCCAATTC-3′ for human LINE-1; 5′-GGTTGAGGTAGTATTTTGTGTG-3′ and 5′-TCCAAAAACTATCAAATTCTCTAAC-3′ for mouse Line-1; 5′-ATTTTGTTGATTAAATAAATTATTATTGGG-3′ and 5′-TAAAACATATCCTCTAATCATTTCTACTCA-3′ for mouse IAP.

### RRBS analysis

The RRBS analysis was performed as described^52^. All sequence data are deposited in the GEO (Gene Expression Omnibus) under accession number GSE136159.

### Flow cytometric analysis of cell cycle

For cell cycle analysis, HeLa cells were fixed in cold 70% ethanol followed by ribonuclease (Tiangen) treatment. The cellular DNA was stained with propidium iodide (Sigma) before analyzed by the BD FACSCalibur flow cytometer. The results were analyzed by ModFit LT 5.0.

### Quantification and statistical analysis

For quantification of protein stability, the western blot bands were quantified by using ImageJ program and shown as relative levels to the control (0 h) in each blot. For histone ubiquitination experiments, the ubiquitinated bands were quantified by using ImageJ, normalized first to the non-ubiquitinated histones (adjusted for loading) and then shown as relative levels to the control. For ChIP-slot blot assay, the slot blot image was quantified by ImageJ and shown as relative levels to control (WT).

All western blots and ChIP-slot blot experiments were performed independently twice or three times. Although the results may not be exactly the same, the overall trends are consistent. All quantified data represent means ± SD for repeats, as referenced in the figure legend. HPLC/RT-PCR/cell growth curve analysis performed at least in three biological replicates (*n* = 3). *P* values<0.05 was considered significant. ****P ≤ 0.0001, ***P ≤ 0.001, **P ≤ 0.01, *P ≤ 0.05.

## Supplementary information

Supplementary Figures

## Supplementary information

**Supplementary Information** accompanies the paper at 10.1038/s41421-020-00188-4.
