# Supplementary material for: USP7 negatively controls global DNA methylation by attenuating ubiquitinated histone-dependent DNMT1 recruitment
Source: Cell Discov. 2020 Aug 24;6:58. doi: 10.1038/s41421-020-00188-4 (PMC7445300; doi:10.1038/s41421-020-00188-4)

**Figure S1**


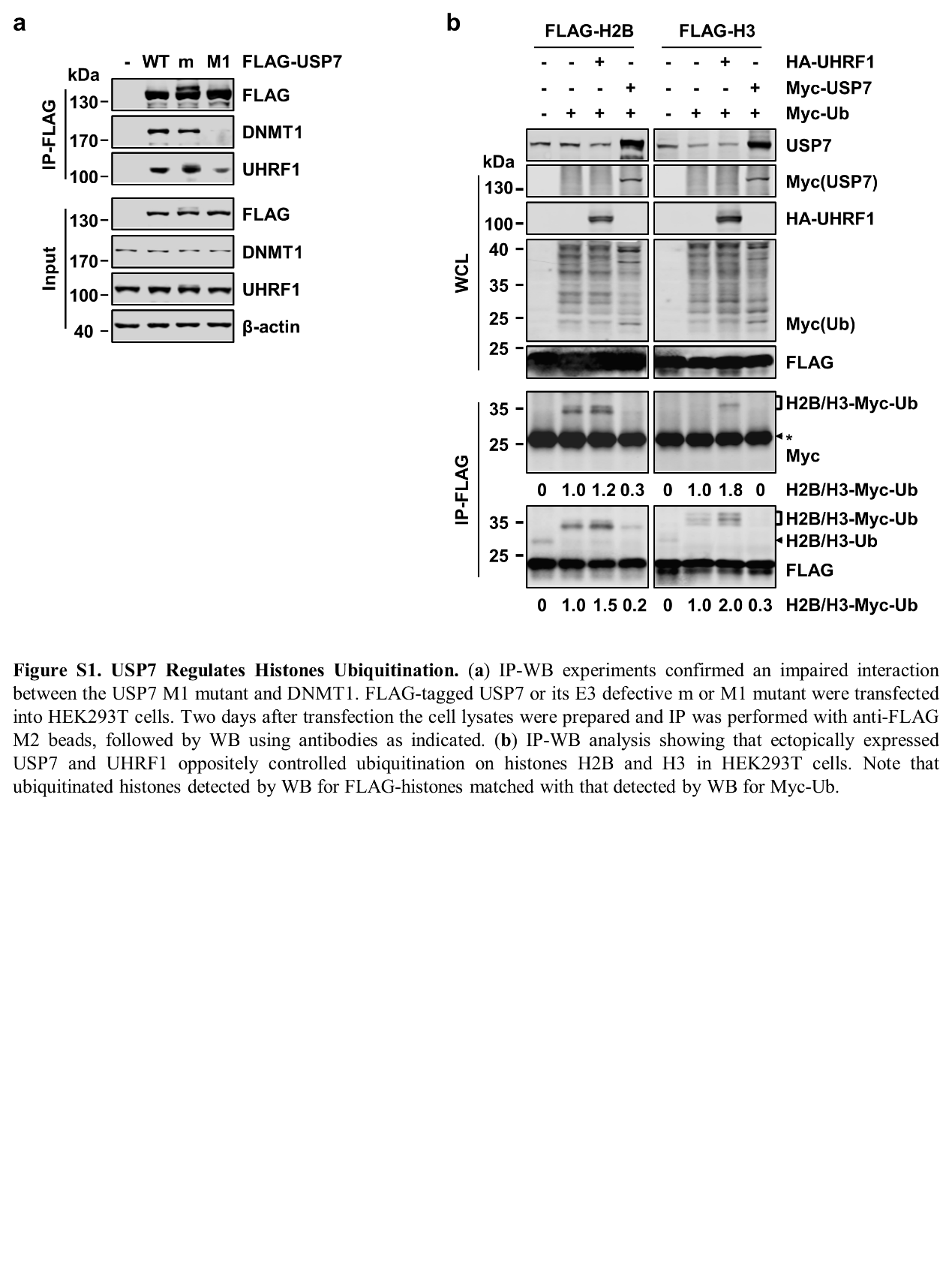


**Figure S1 USP7 Regulates Histones Ubiquitination.** (**a**) IP-WB experiments confirmed an impaired interaction between the USP7 M1 mutant and DNMT1. FLAG-tagged USP7 or its E3 defective m or M1 mutant were transfected into HEK293T cells. Two days after transfection the cell lysates were prepared and IP was performed with anti-FLAG M2 beads, followed by WB using antibodies as indicated. (**b**) IP-WB analysis showing that ectopically expressed USP7 and UHRF1 oppositely controlled ubiquitination on histones H2B and H3 in HEK293T cells. Note that ubiquitinated histones detected by WB for FLAG-histones matched with that detected by WB for Myc-Ub.

**Figure S2**


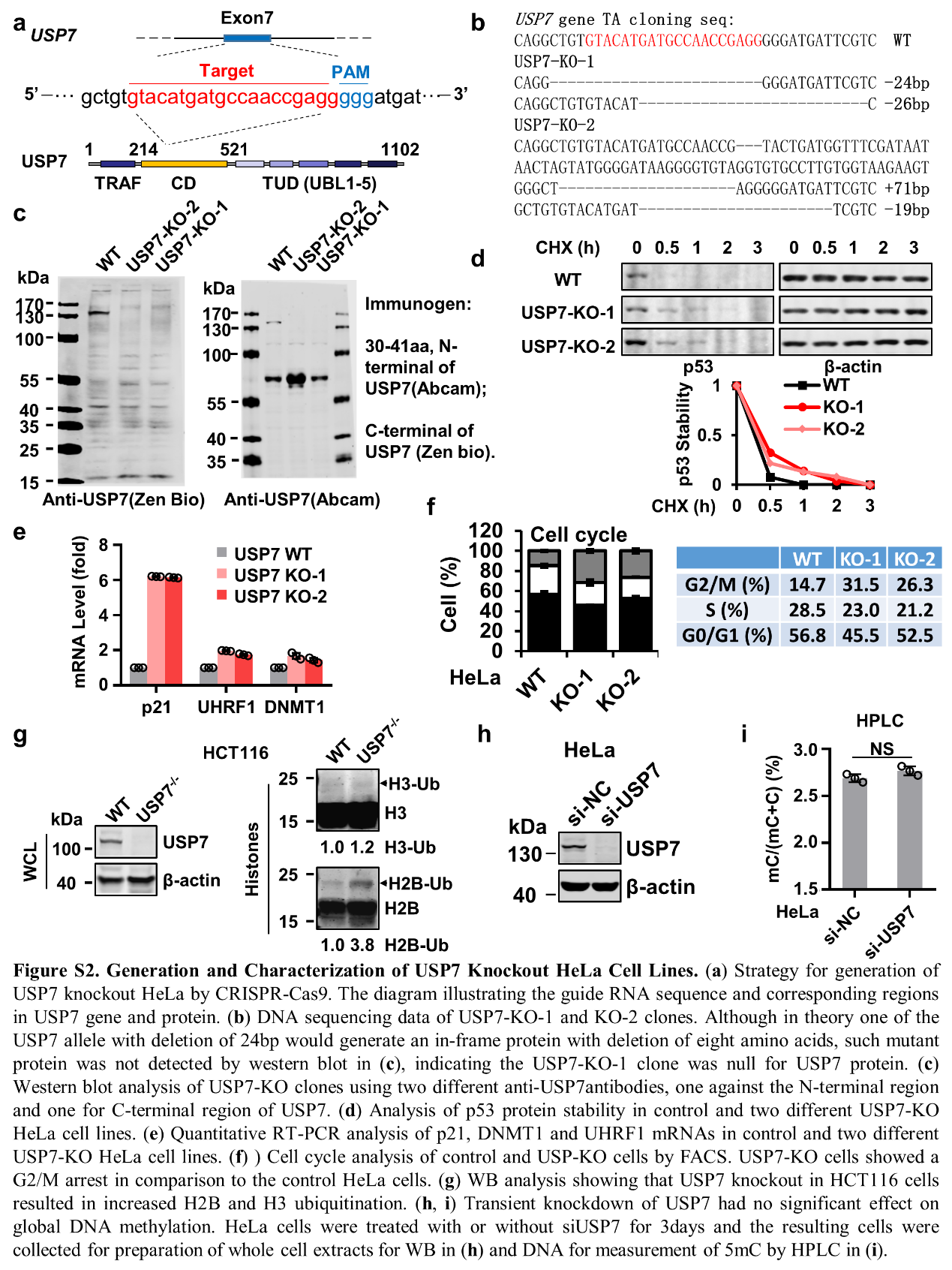


**Figure S2 Generation and Characterization of USP7 Knockout HeLa Cell Lines.** (**a**) Strategy for generation of USP7 knockout HeLa by CRISPR-Cas9. The diagram illustrating the guide RNA sequence and corresponding regions in USP7 gene and protein. (**b**) DNA sequencing data of USP7-KO-1 and KO-2 clones. Although in theory one of the USP7 allele with deletion of 24bp would generate an in-frame protein with deletion of eight amino acids, such mutant protein was not detected by western blot in (**c**), indicating the USP7-KO-1 clone was null for USP7 protein. (**c**) Western blot analysis of USP7-KO clones using two different anti-USP7antibodies, one against the N-terminal region and one for C-terminal region of USP7. (**d**) Analysis of p53 protein stability in control and two different USP7-KO HeLa cell lines. (**e**) Quantitative RT-PCR analysis of p21, DNMT1 and UHRF1 mRNAs in control and two different USP7-KO HeLa cell lines. (**f**) ) Cell cycle analysis of control and USP-KO cells by FACS. USP7-KO cells showed a G2/M arrest in comparison to the control HeLa cells. (**g**) WB analysis showing that USP7 knockout in HCT116 cells resulted in increased H2B and H3 ubiquitination. (**h**, **i**) Transient knockdown of USP7 had no significant effect on global DNA methylation. HeLa cells were treated with or without siUSP7 for 3days and the resulting cells were collected for preparation of whole cell extracts for WB in (**h**) and DNA for measurement of 5mC by HPLC in (**i**).

**Figure S3**


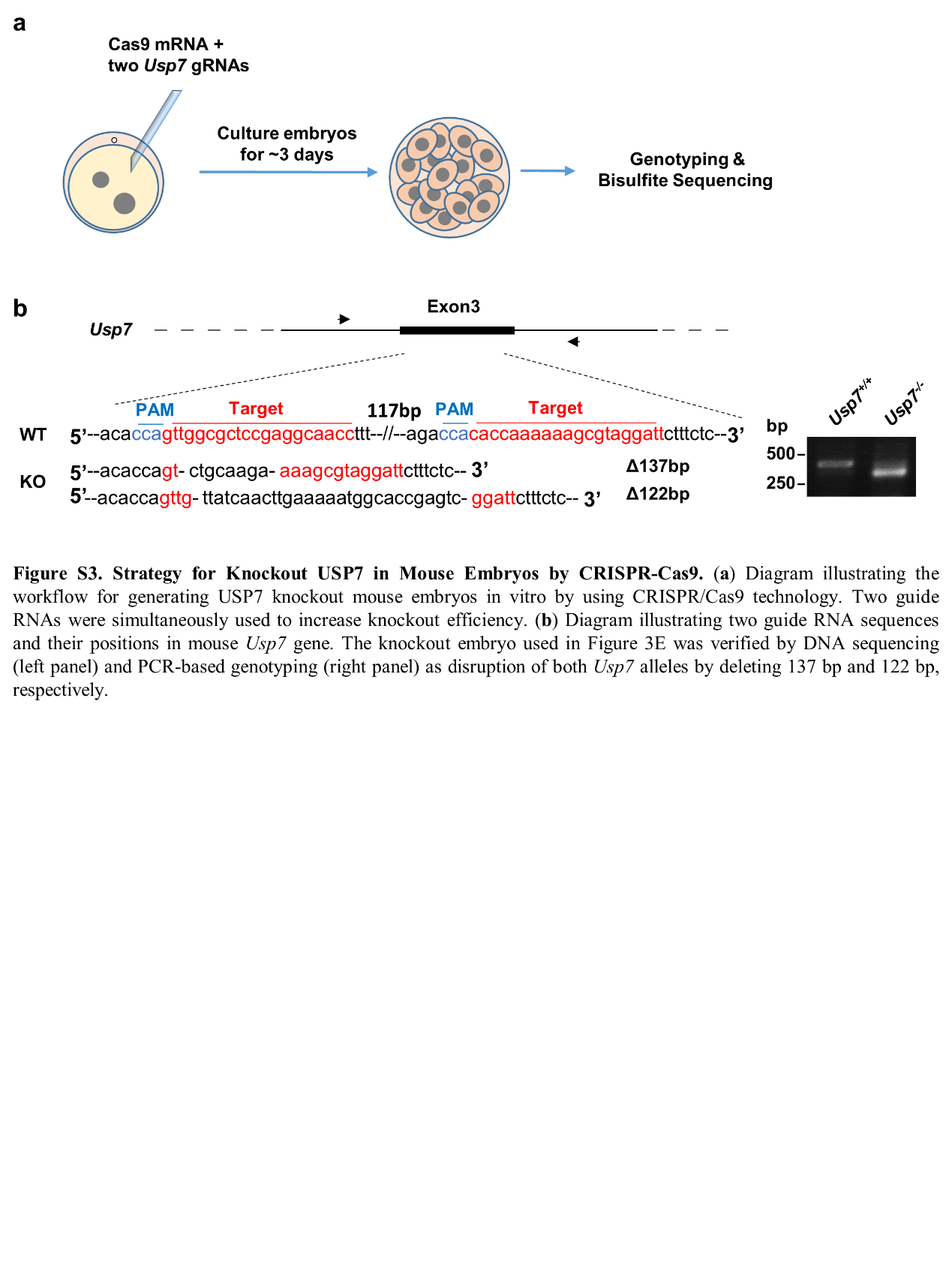


**Figure S3 Strategy for Knockout USP7 in Mouse Embryos by CRISPR-Cas9.** (**a**) Diagram illustrating the workflow for generating USP7 knockout mouse embryos in vitro by using CRISPR/Cas9 technology. Two guide RNAs were simultaneously used to increase knockout efficiency. (**b**) Diagram illustrating two guide RNA sequences and their positions in mouse *Usp7* gene. The knockout embryo used in Figure 3E was verified by DNA sequencing (left panel) and PCR-based genotyping (right panel) as disruption of both *Usp7* alleles by deleting 137 bp and 122 bp, respectively.

**Figure S4**


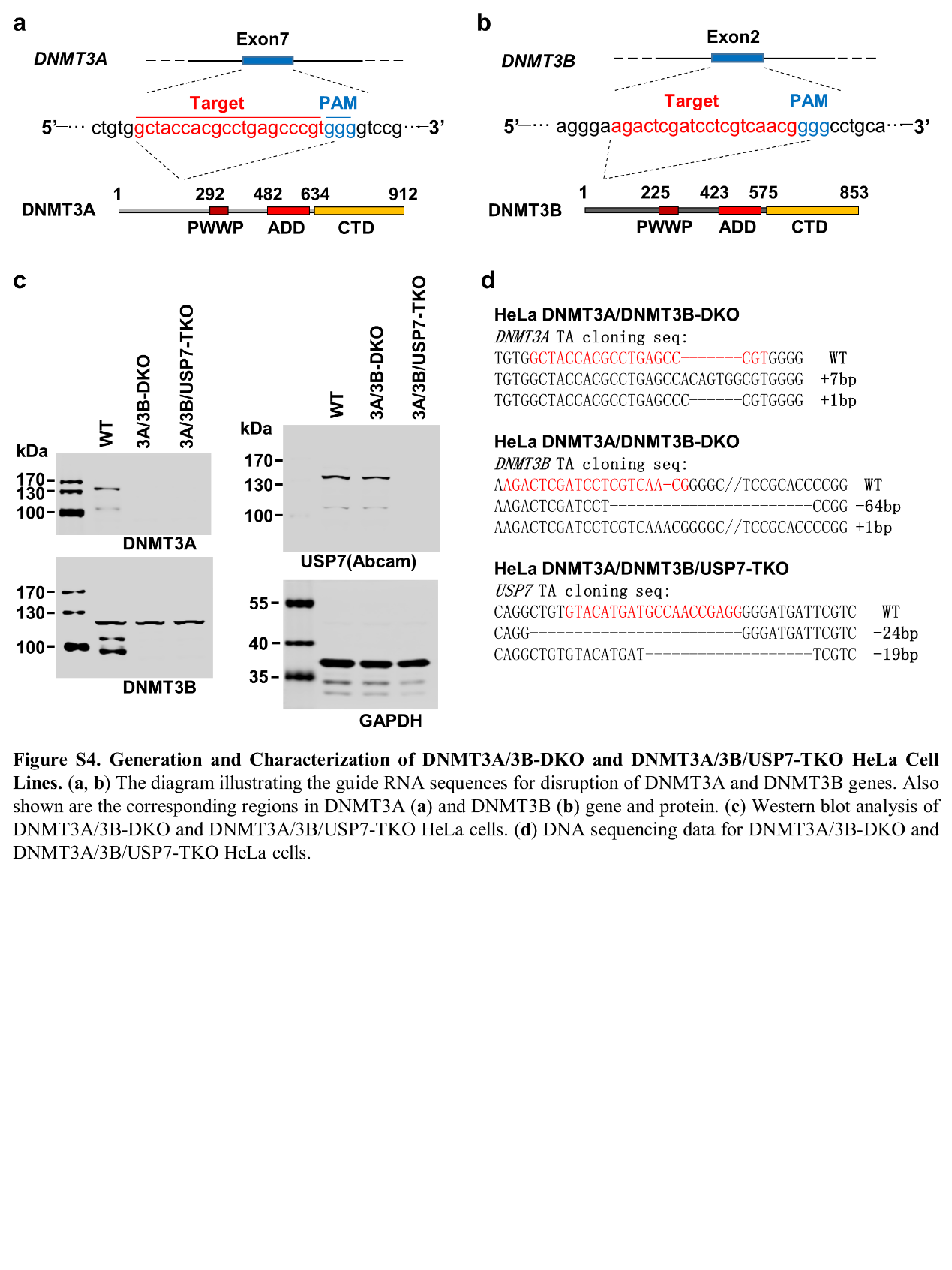


**Figure S4 Generation and Characterization of DNMT3A/3B-DKO and DNMT3A/3B/USP7-TKO HeLa Cell Lines.** (**a**, **b**) The diagram illustrating the guide RNA sequences for disruption of DNMT3A and DNMT3B genes. Also shown are the corresponding regions in DNMT3A (**a**) and DNMT3B (**b**) gene and protein. (**c**) Western blot analysis of DNMT3A/3B-DKO and DNMT3A/3B/USP7-TKO HeLa cells. (**d**) DNA sequencing data for DNMT3A/3B-DKO and DNMT3A/3B/USP7-TKO HeLa cells.

**Figure S5**

**RRBS data**


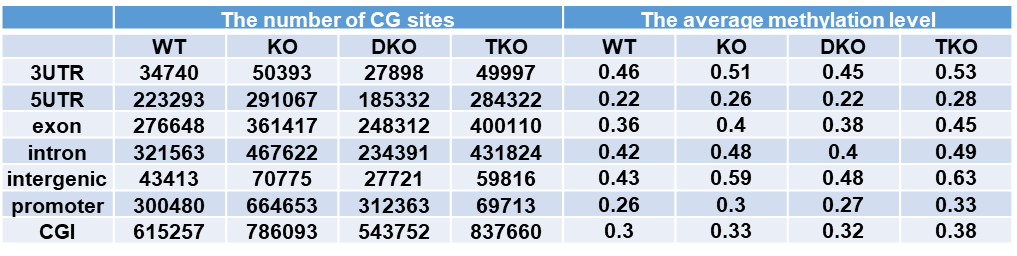


**Figure S5 The RRBS Sequencing Information for Different Genetic Elements.**

**Figure S6**


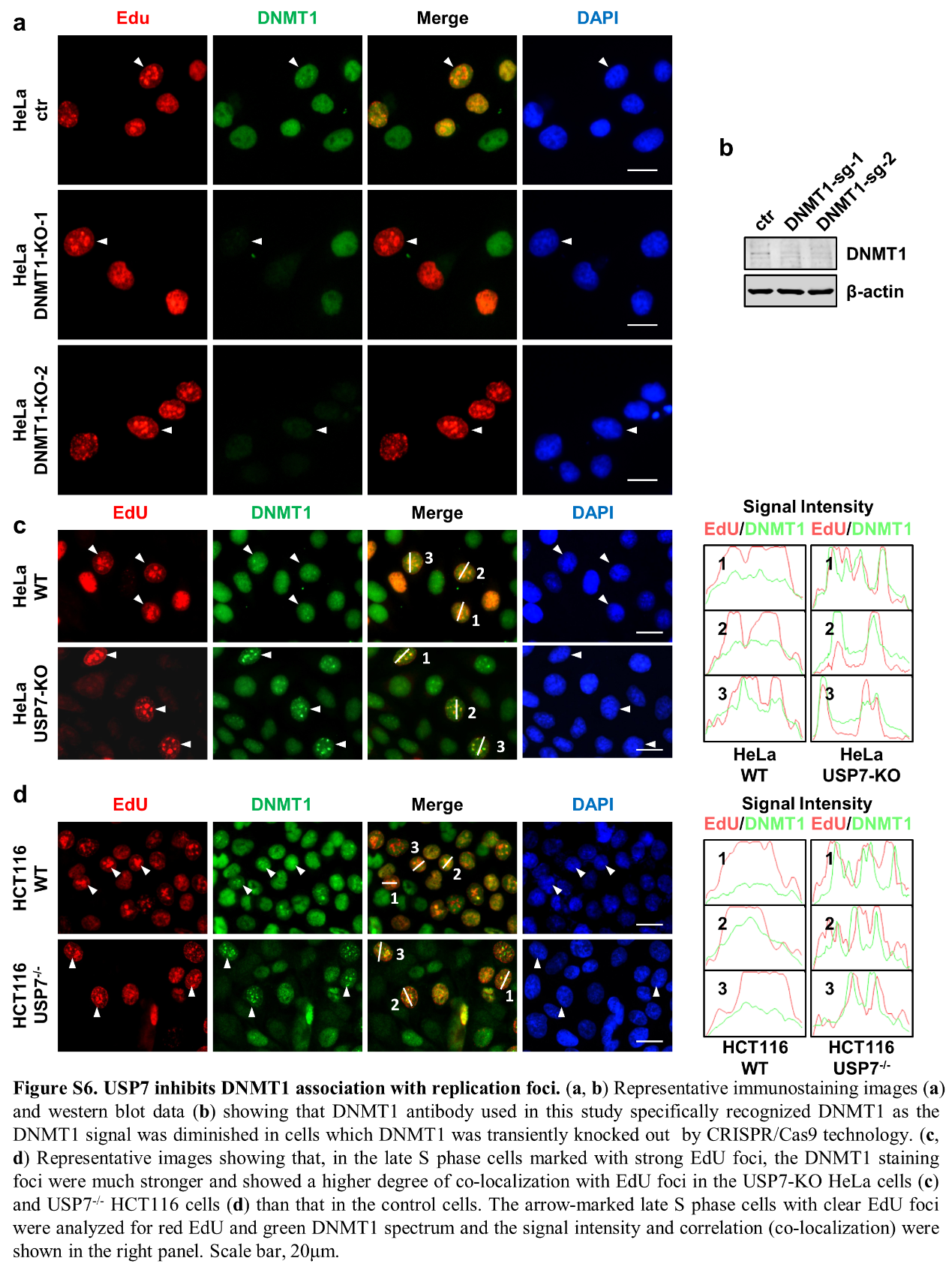


**Figure S6 USP7 inhibits DNMT1 association with replication foci.** (**a**, **b**) Representative immunostaining images (**a**) and western blot data (**b**) showing that DNMT1 antibody used in this study specifically recognized DNMT1 as the DNMT1 signal was diminished in cells which DNMT1 was transiently knocked out by CRISPR/Cas9 technology. (**c**, **d**) Representative images showing that, in the late S phase cells marked with strong EdU foci, the DNMT1 staining foci were much stronger and showed a higher degree of co-localization with EdU foci in the USP7-KO HeLa cells (**c**) and USP7-/- HCT116 cells (**d**) than that in the control cells. The arrow-marked late S phase cells with clear EdU foci were analyzed for red EdU and green DNMT1 spectrum and the signal intensity and correlation (co-localization) were shown in the right panel. Scale bar, 20μm.

**Figure S7**


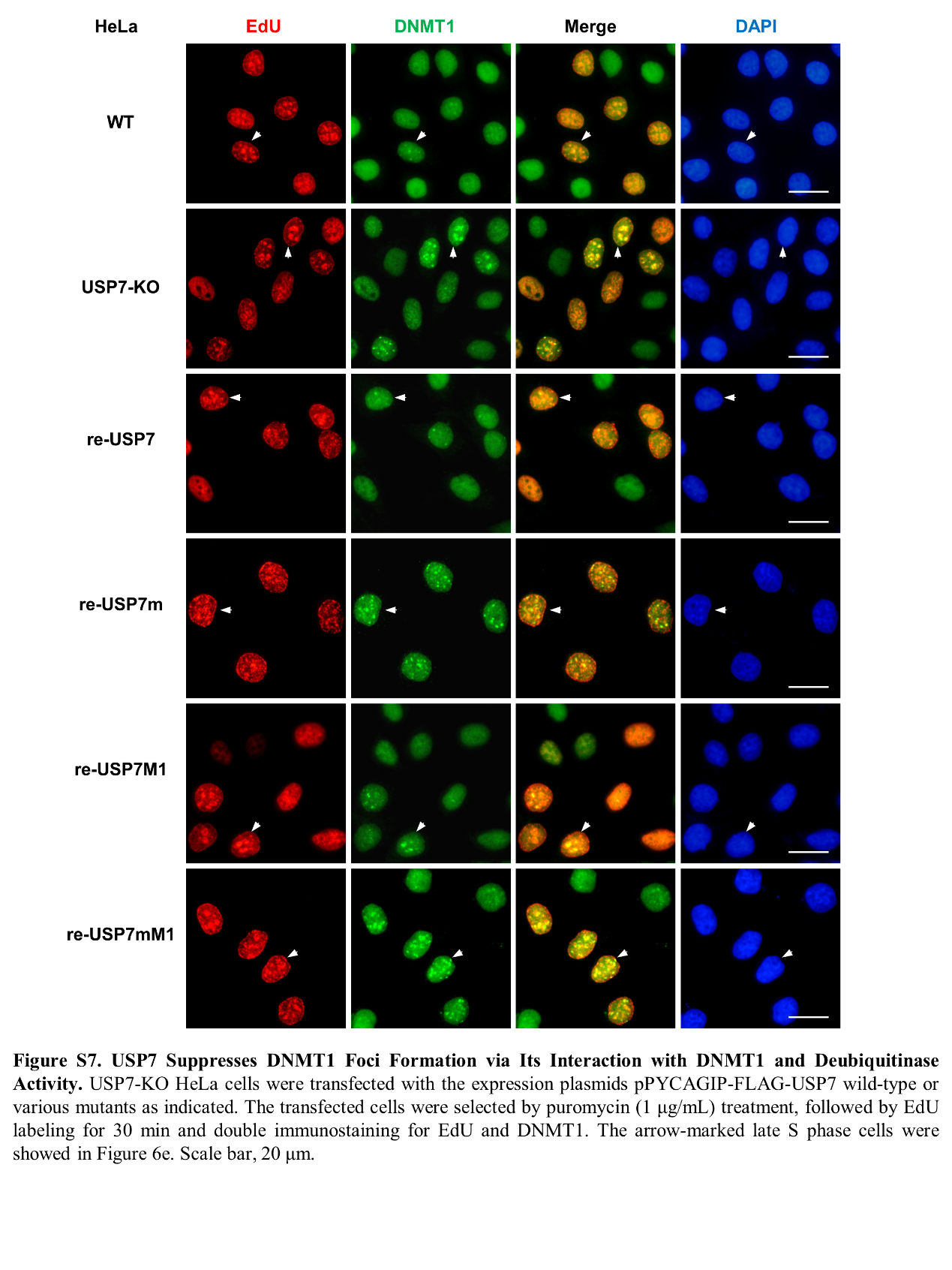


**Figure S7 USP7 Suppresses DNMT1 Foci Formation via Its Interaction with DNMT1 and Deubiquitinase Activity.** USP7-KO HeLa cells were transfected with the expression plasmids pPYCAGIP-FLAG-USP7 wild-type or various mutants as indicated. The transfected cells were selected by puromycin (1 μg/mL) treatment, followed by EdU labeling for 30 min and double immunostaining for EdU and DNMT1. The arrow-marked late S phase cells were showed in Figure 6e. Scale bar, 20 μm.

**Figure S8**


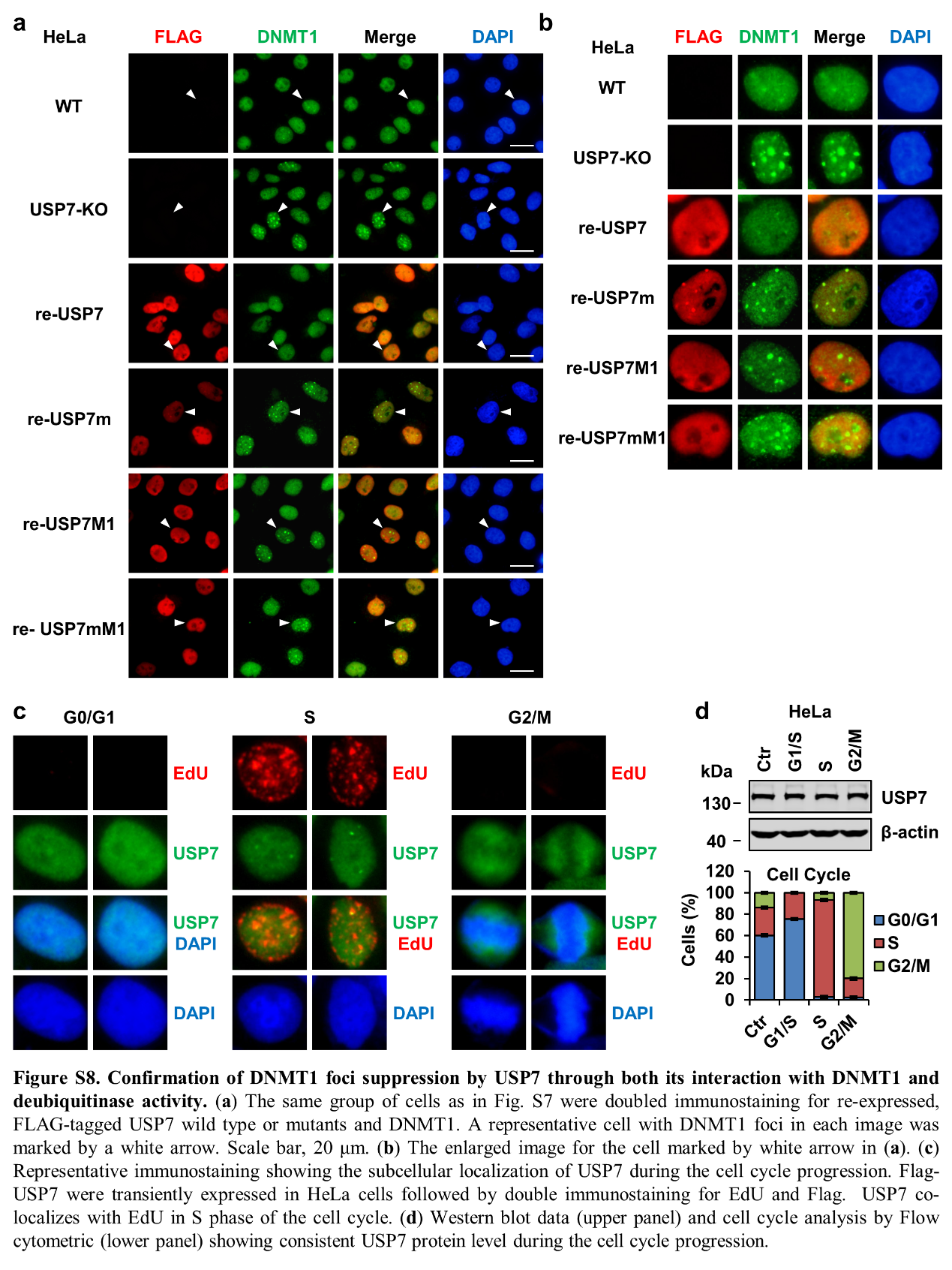


**Figure S8 Confirmation of DNMT1 foci suppression by USP7 through both its interaction with DNMT1 and deubiquitinase activity.** (**a**) The same group of cells as in Fig. S7 were doubled immunostaining for re-expressed, FLAG-tagged USP7 wild type or mutants and DNMT1. A representative cell with DNMT1 foci in each image was marked by a white arrow. Scale bar, 20 μm. (**b**) The enlarged image for the cell marked by white arrow in (**a**). (**c**) Representative immunostaining showing the subcellular localization of USP7 during the cell cycle progression. Flag-USP7 were transiently expressed in HeLa cells followed by double immunostaining for EdU and Flag. USP7 co-localizes with EdU in S phase of the cell cycle. (**d**) Western blot data (upper panel) and cell cycle analysis by Flow cytometric (lower panel) showing consistent USP7 protein level during the cell cycle progression.

**Figure S9**

**Uncropped blots used for this study.**


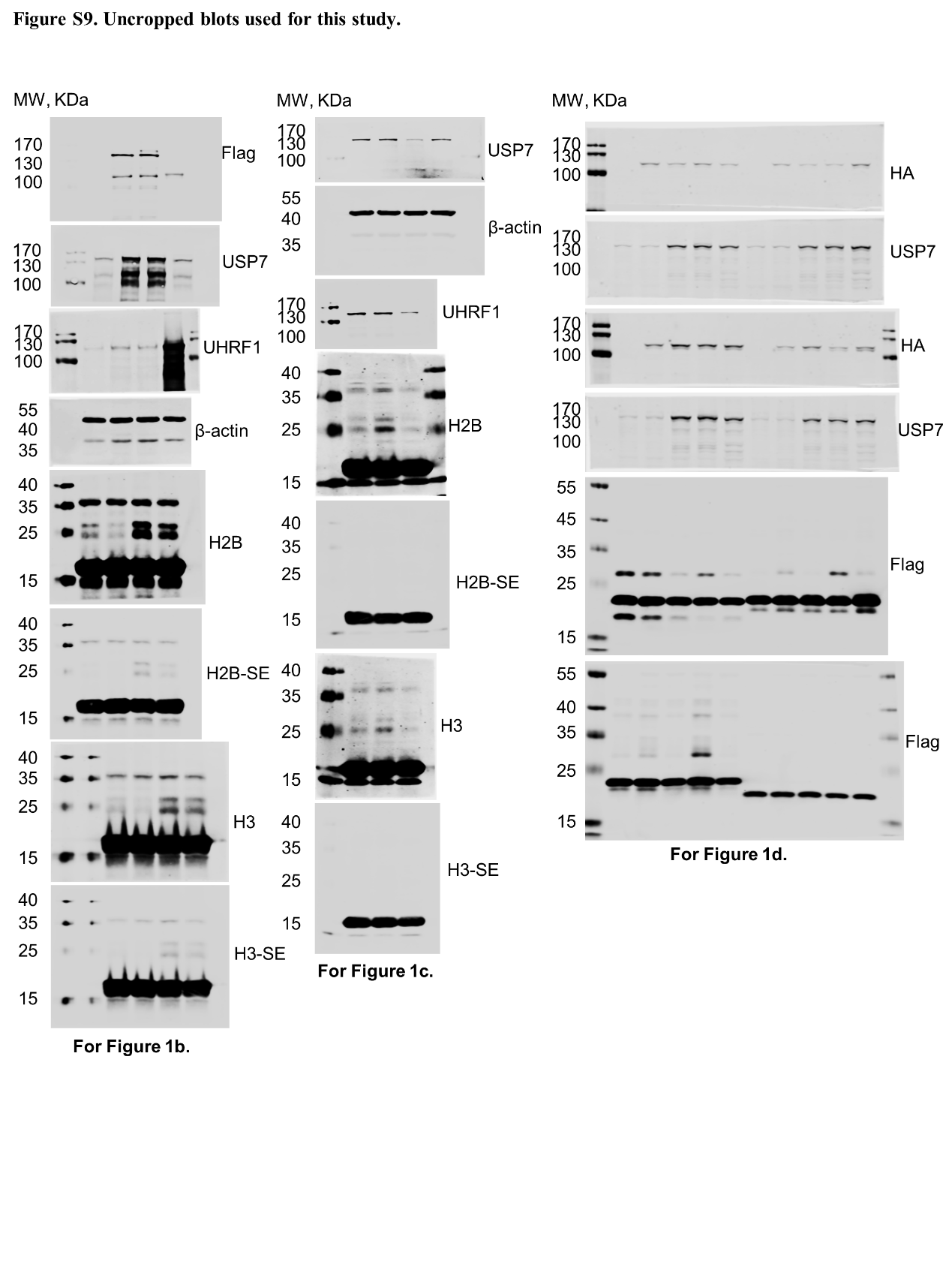


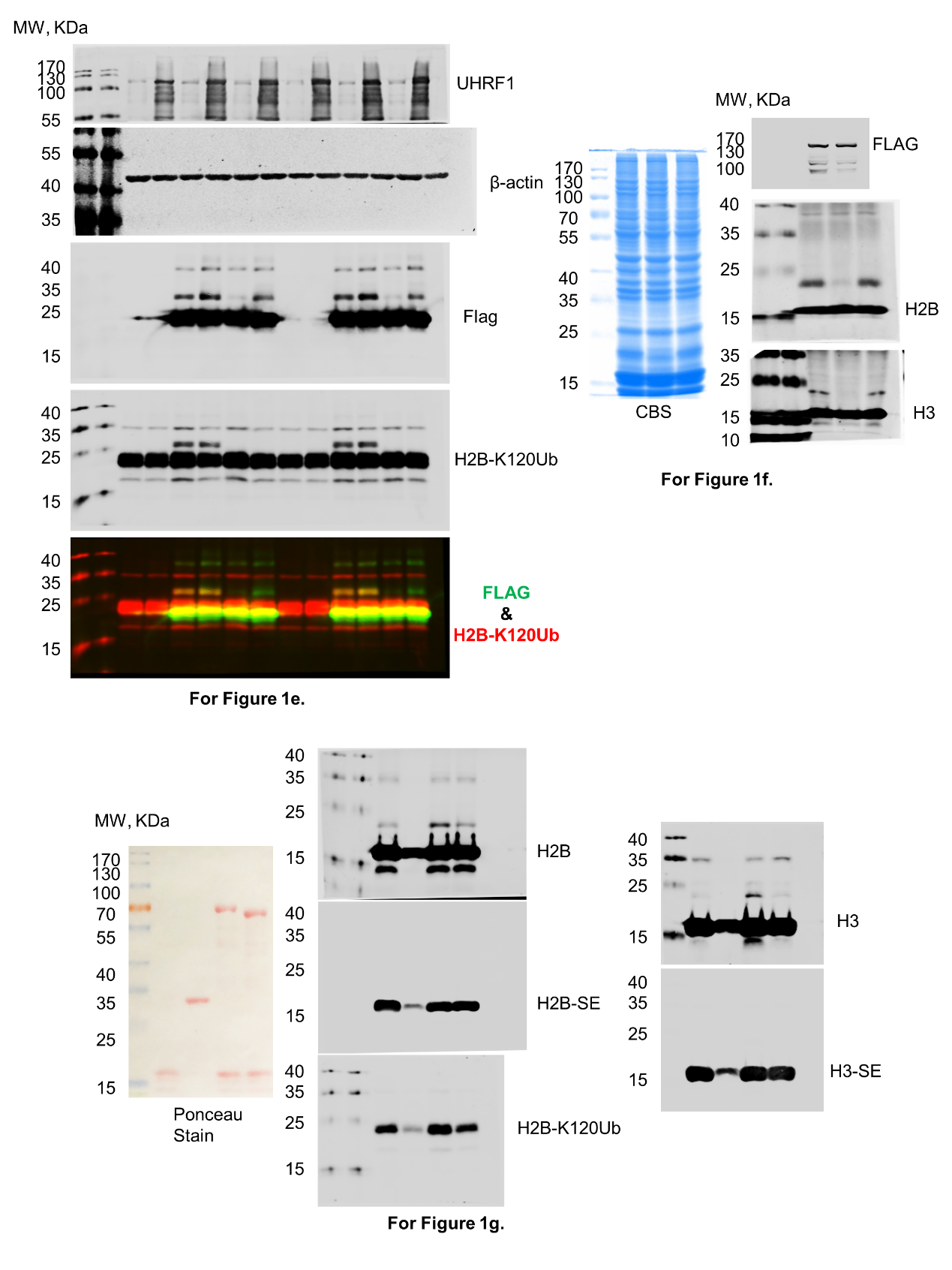


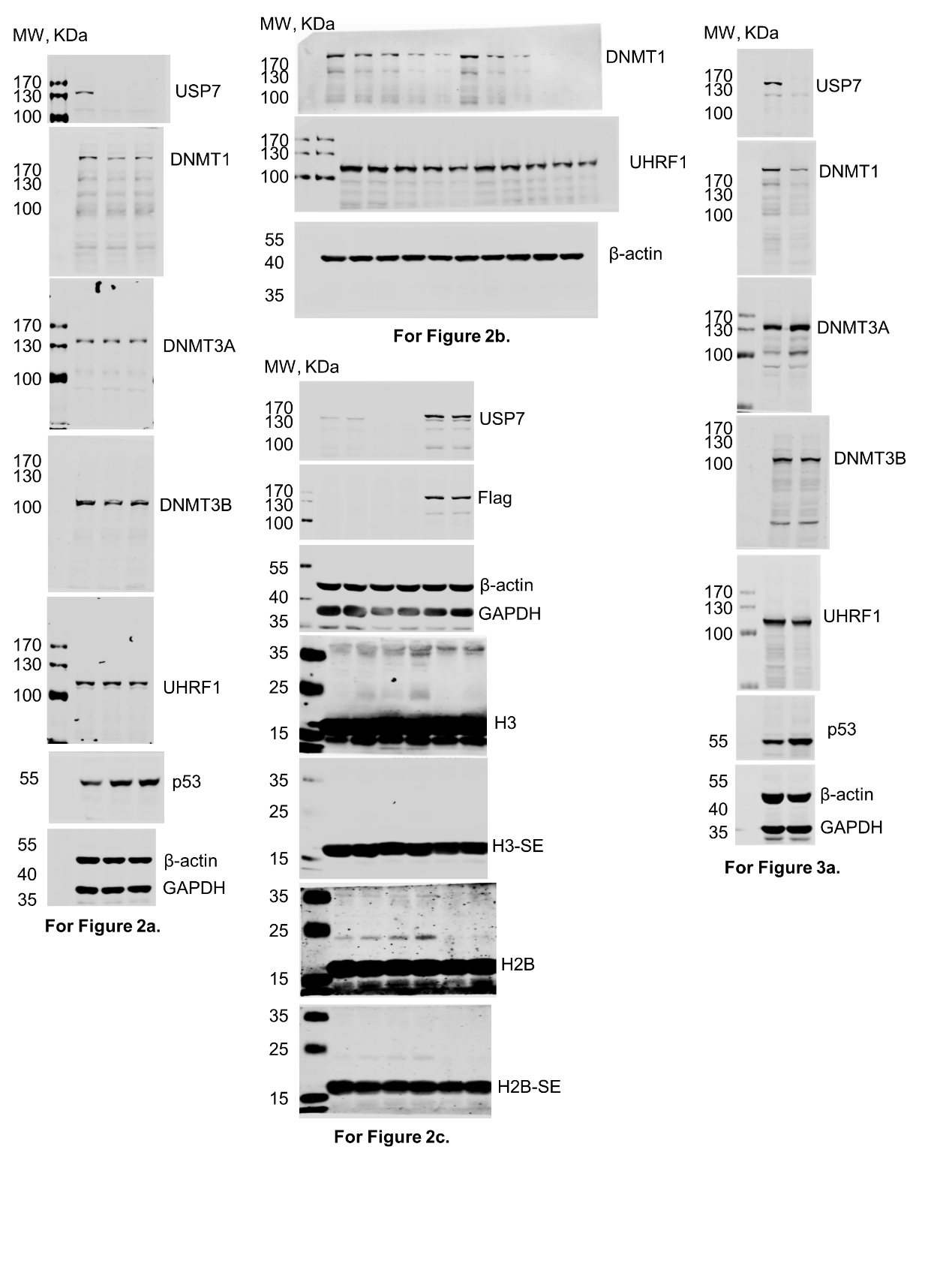


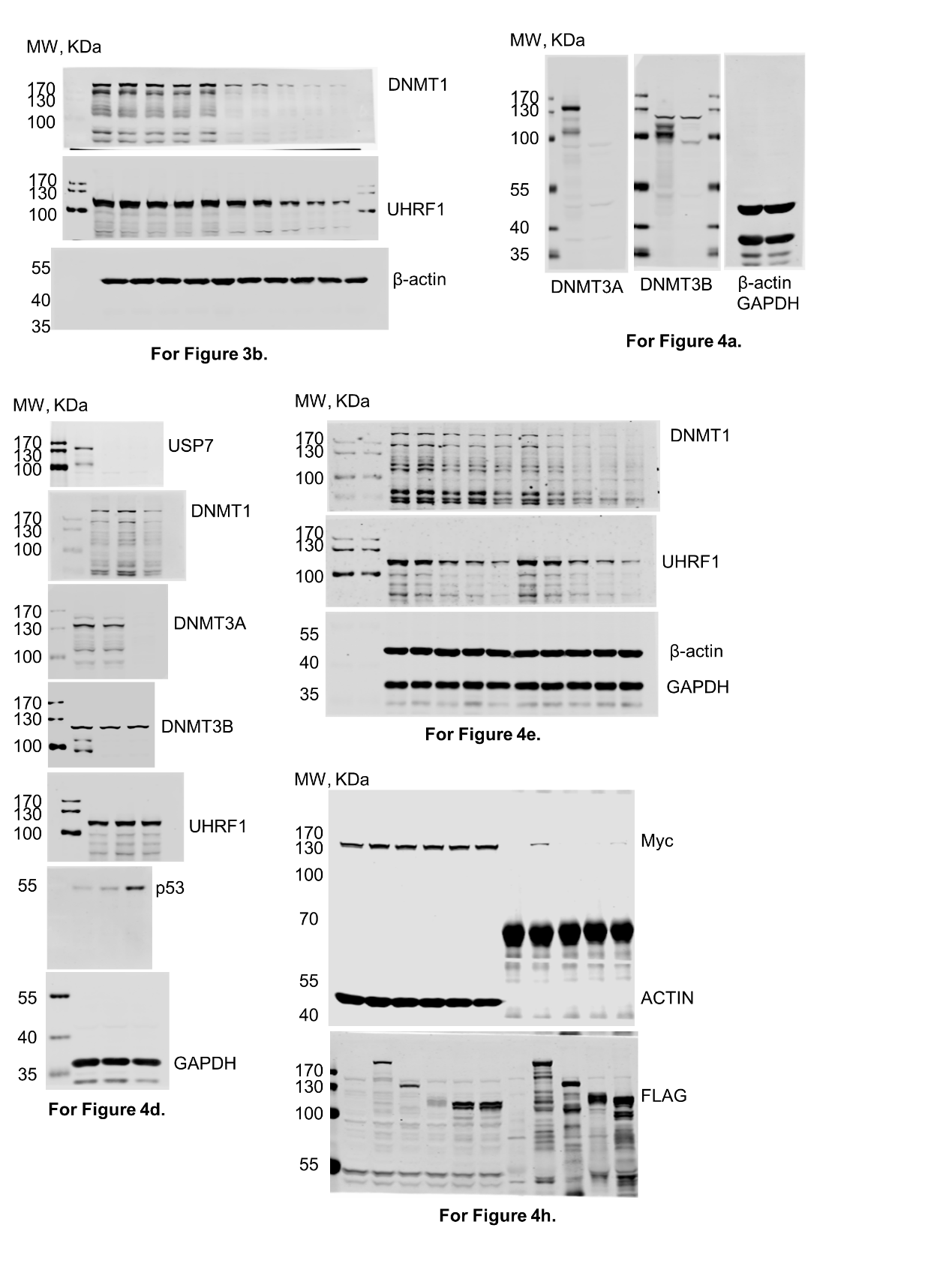


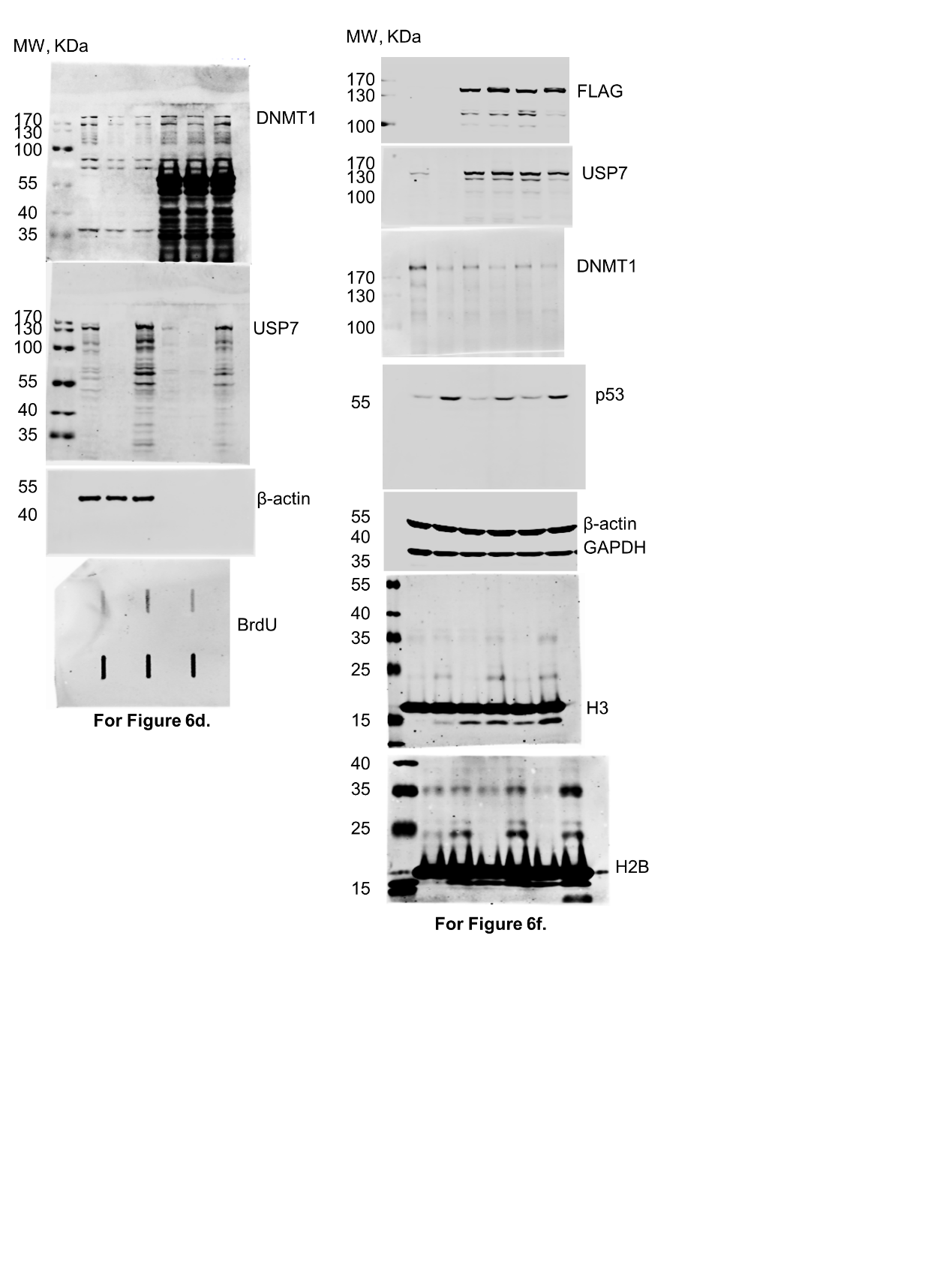


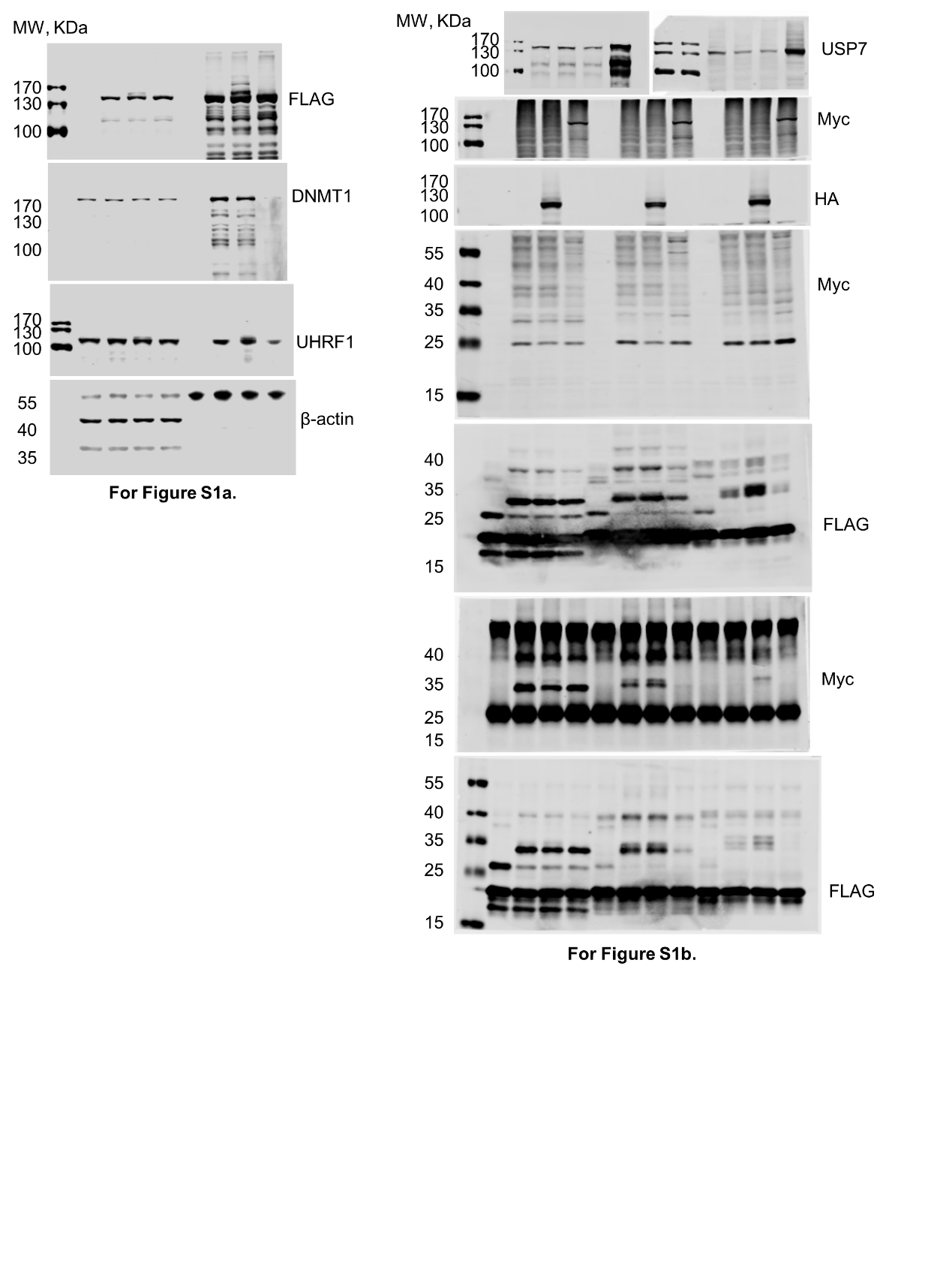


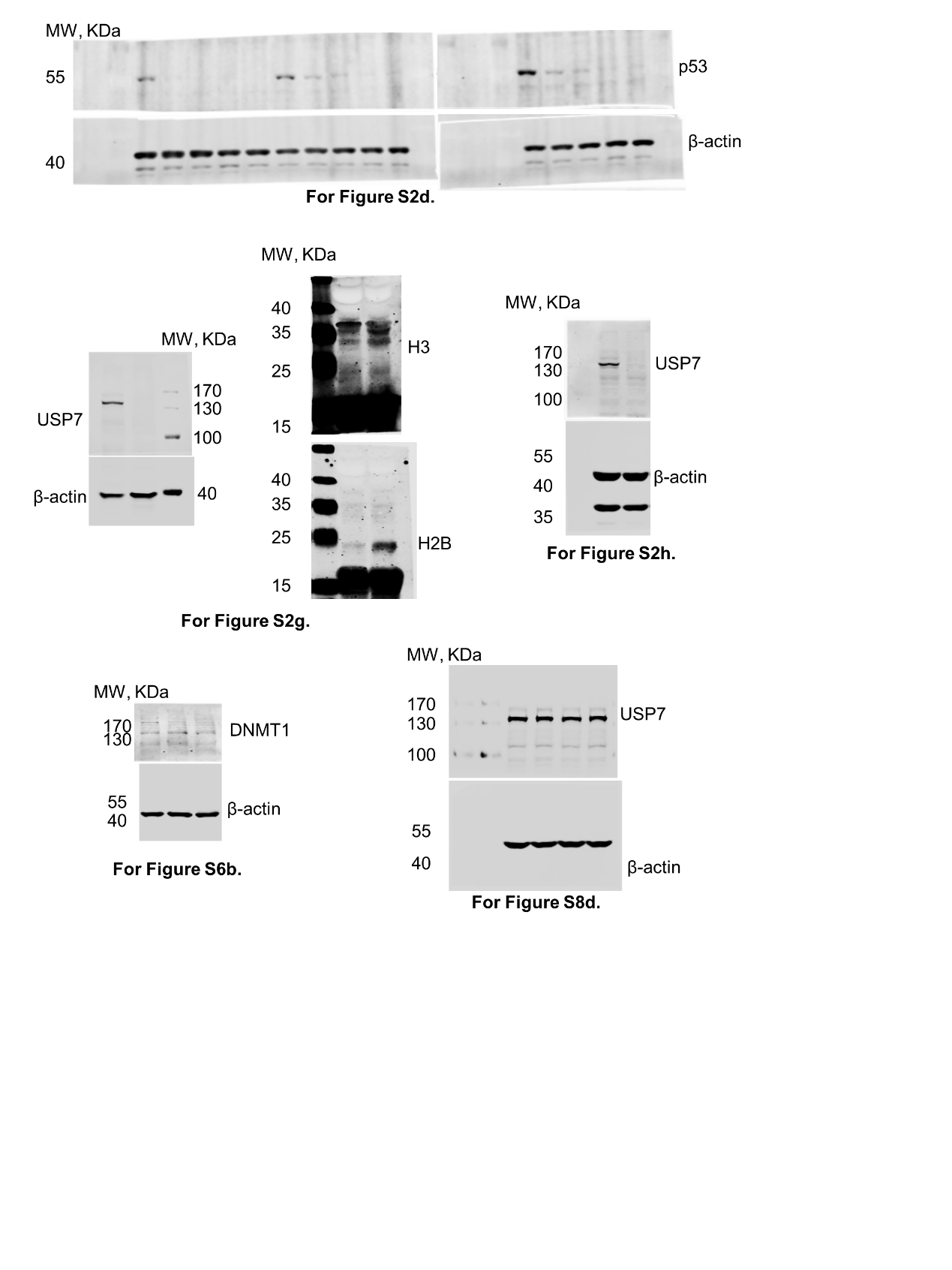

Supplement: Supplementary file 1 — Supplementary Figures [file 41421_2020_188_MOESM1_ESM.docx]
